# A Rare Allele of *ST5* From Wild Rice Enhances Salt Tolerance in Rice

**DOI:** 10.1002/advs.202516159

**Published:** 2026-05-28

**Authors:** Meng Xing, Jingfen Huang, Qiaoling Yuan, Ziyi Yang, Yanyan Wang, Mingchao Zhao, Yamin Nie, Rui Xu, Hongge Qian, Wenxi Chen, Qiaoling Zhang, Qi Du, Leiyue Geng, Yapeng Li, Ziyi Chen, Shizhuang Wang, Like Lou, Haiyuan Peng, Chongke Zheng, Xianzhi Xie, Xiaoming Zheng, Lifang Zhang, Lianguang Shang, Jiaqiang Sun, Qian Qian, Qingwen Yang, Weihua Qiao

**Affiliations:** ^1^ State Key Laboratory of Crop Gene Resources and Breeding Institute of Crop Sciences Chinese Academy of Agricultural Sciences Beijing China; ^2^ National Nanfan Research Institute Chinese Academy of Agricultural Sciences Sanya China; ^3^ State Key Laboratory of Genome and Multi‐omics Technologies Shenzhen Branch Guangdong Laboratory for Lingnan Modern Agriculture Genome Analysis Laboratory of the Ministry of Agriculture and Rural Affairs Agricultural Genomics Institute at Shenzhen Chinese Academy of Agricultural Sciences Shenzhen China; ^4^ Cereal Crop Institute Hainan Academy of Agricultural Sciences Haikou China; ^5^ Jining Academy of Agricultural Sciences Jining Shandong China; ^6^ Institute of Coastal Agriculture Hebei Academy of Agriculture and Forestry Sciences Tangshan China; ^7^ China National Rice Research Institute Hangzhou China; ^8^ Institute of Wetland Agriculture and Ecology, Shandong Academy of Agricultural Sciences Jinan China; ^9^ Yazhouwan National Laboratory Sanya China

**Keywords:** Na^+^/K^+^ homeostasis, rare allele, salt tolerance, *ST5*, wild rice

## Abstract

Soil salinity critically impairs global rice productivity, necessitating the exploration of salt‐tolerant genetic resources in wild rice (*Oryza rufipogon*). Here, we identified a C2H2 transcription factor, ST5, from wild rice using a chromosome segment substitution line population. Functional analysis reveals that *ST5* negatively regulates rice salt tolerance. A 36‐bp insertion in the *ST5^W^* promoter harbors two W‐box motifs, transcription factor OsWRKY80 binds to this insertion and represses *ST5^W^* expression. This repression reduces *ST5^W^* expression, alleviating its negative regulation on the downstream genes *OsCPK4*, which are pivotal for maintaining Na^+^/K^+^ homeostasis under salinity stress. Notably, the *ST5^W^* allele is exclusively present in a few of *O. rufipogon* accessions and absent in all cultivated rice varieties. Field trials demonstrate *ST5^W^* significantly improves grain yield across diverse genetic backgrounds under saline field conditions. Our work provides both an underexploited genetic resource and molecular insights for breeding salt‐tolerant rice varieties to address soil salinization challenges.

## Graphical abstract

A novel salt‐tolerance gene *ST5^W^*, characterized by a unique 36‐bp promoter insertion, is found exclusively in a small subset of *Oryza rufipogon* and is absent in cultivated rice. Field trials confirm *ST5^W^* significantly enhances rice yield across diverse genetic backgrounds under saline conditions. This underexploited genetic resource, combined with its elucidated regulatory mechanism, provides valuable insights for breeding salt‐tolerant rice.

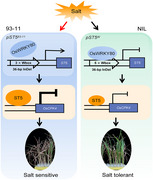

## 1 Introduction

According to a recent FAO report (2024), approximately 1,381 million hectares (10.7% of the world's arable land) are affected by salinity, with substantial crop yield losses observed in these areas [1]. Soil salinity reduces crop yields by imposing both osmotic and ionic stresses on plants [2, 3], especially for cultivated rice (*Oryza sativa*), which is considered the most salt‐sensitive crop of all cereals [4]. Rice productivity begins to decline when soil salinity levels exceed 3 dS m^−^^1^ (as measured by the electrical conductivity of the soil extract, ECe), with each additional 1 dS m^−^^1^ increase resulting in a 12% yield reduction [4]. Salt tolerance in rice is governed by complex quantitative traits involving multiple genes and intricate molecular mechanisms [5]. Under salt stress, rice regulates tolerance primarily by maintaining ionic homeostasis. High cytosolic K^+^ levels and a favorable K^+^/Na^+^ ratio in photosynthetic cells are critical for plant survival under salinity [6, 7]. Transcription factor (TF) families, including C2H2‐type zinc finger [8, 9, 10, 11], and WRKY, are involved in ionic homeostasis and salt tolerance. WRKY factors bind W‐box elements in target gene promoters to activate or repress transcription, and their functions vary significantly in different species [12, 13, 14, 15]. While research continues to identify novel functional genes and their interactions in salinity tolerance, this remains a challenging area of investigation.

Wild rice species represent a valuable genetic reservoir, harboring extensive diversity including rare alleles that often confer enhanced adaptability and strong stress resistance [16, 17]. Although these alleles typically occur at low frequencies in natural populations, their identification and utilization are crucial for rice genetic improvement [18, 19]. Notably, many salt‐responsive genes from wild relatives are associated with undesirable agronomic traits, limiting their practical utilization. To date, lots of genes associated with biotic and abiotic stress resistance, yield‐related traits, and other agronomically important characteristics have been identified across 13 wild rice species, including *LEA12* [20], *GL12* [21], *STG5* [22], and *OsMADS56* [23], which are involved in salt tolerance. However, none of these genes have been reported to contain unique alleles from wild rice that could be exploited for crop improvement.

In this study, we identified a novel rare allele *ST5^W^* conferring salt tolerance in the wild rice species *O. rufipogon*. Our findings demonstrate that a 36‐bp insertion in the *ST5^W^* promoter mediates its repression by OsWRKY80, while ST5 itself directly downregulates *OsCPK4* expression to modulate Na^+^/K^+^ homeostasis. This beneficial allele appears exclusively in certain *O. rufipogon* accessions and is absent from cultivated rice and other wild species. Introgression of *ST5^W^* into modern rice cultivars significantly improved grain yield under saline conditions. Our work not only provides a valuable genetic resource for addressing the increasing challenge of soil salinization in agriculture but also advances our understanding of the mechanism of salt tolerance in rice.

## 2 Results

### 2.1 Identification of Salt Tolerance Locus *ST5* From Wild Rice

**FIGURE 1:**
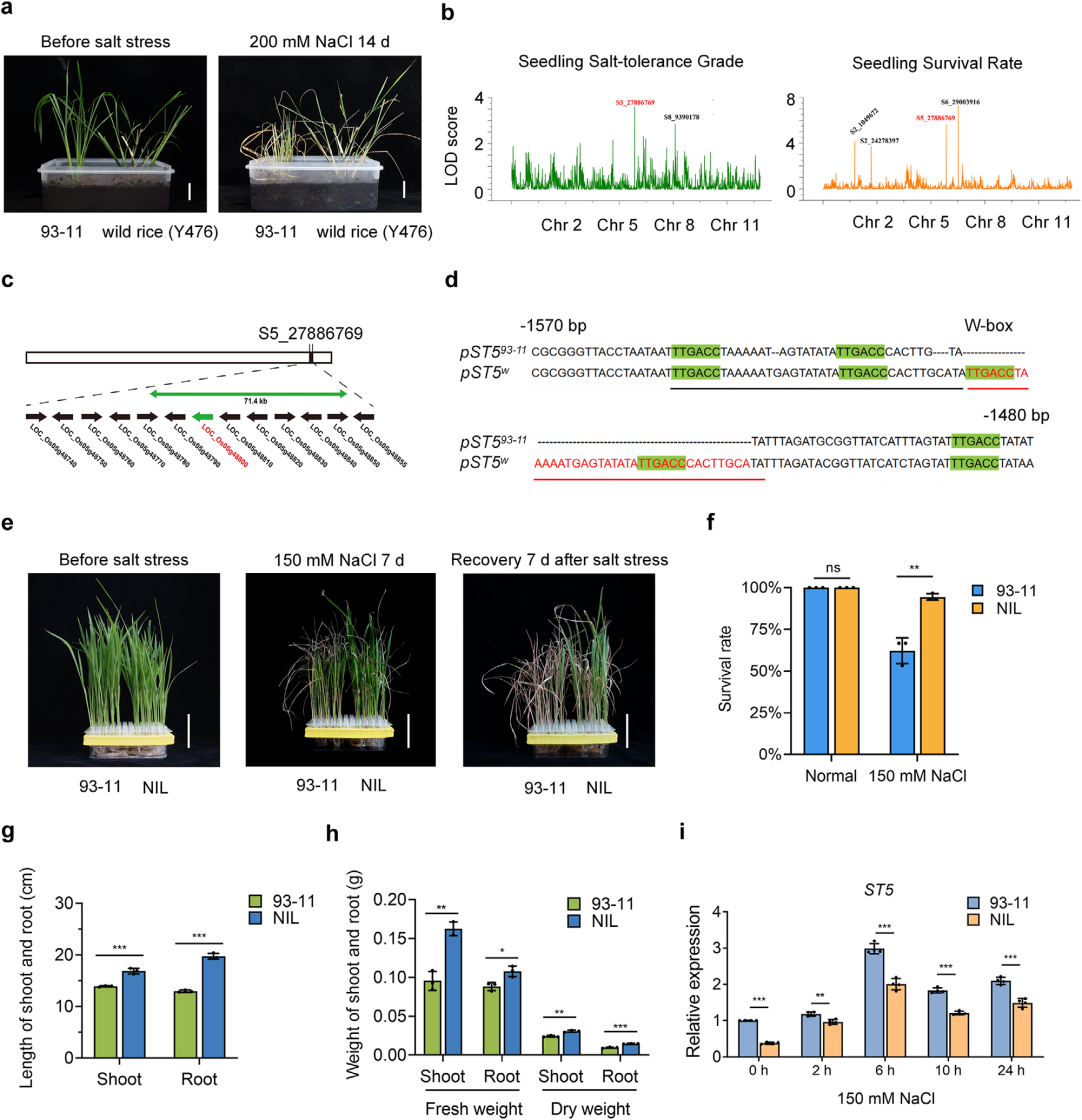
Map‐based cloning and identification of the salt tolerance gene *ST5* using a wild rice CSSL population. a) Salt tolerance phenotype of 93‐11 and wild rice (accession number Y476). Scale bars = 5 cm. b) Mapping of QTLs related to different salt tolerance index using wild rice CSSL population. The S5_27886769 locus was associated with both Seedling Salt‐tolerant Grade and Seedling Survival Rate. The x‐axis showed the 12 chromosomes, and the y‐axis showed the logarithm of odds (LOD) score. c) The *qST5* locus was located to a 71.4‐kb interval near S5_27886769 markers on Chr.5. The black arrows represent 13 open reading frames of candidate genes, and the *LOC_Os05g48800* was marked in red font. d) Comparison of promoters of *ST5* alleles between 93‐11 (*pST5^93‐11^*) and wild rice (*pST5^W^*). The 36‐bp InDel contains putative W‐boxes (highlighted by a green box). The single‐nucleotide polymorphisms are capitalized and shown in red, and deletions are indicated by dashed lines. In *pST5^W^*, the sequence above the red solid line is a copy of the sequence above the black solid line. e‐h) Salt tolerance phenotype of 93‐11 and the NIL. The survival rate (f), length of shoot and root (g), and fresh and dry weight of shoot and root (h) were determined after a seven‐day recovery period. Scale bars = 5 cm. i) The relative expression of *ST5* in 93‐11 and the NIL with different treatment times in 150 mM NaCl solution. Data are means ± SD (*n* = 3 biological replicates). ^*^, *P* < 0.05; ^**^, *P* < 0.01; ^***^, *P* < 0.001; ns, no significant difference. Two‐tailed Student's *t*‐test.

In our previous study [19, 24], a set of chromosome segment substitution lines (CSSLs) was constructed with the cultivated rice *indica* variety ‘93‐11’ as the recurrent parent and wild rice (*O. rufipogon* Griff.) accession Y476 as the donor parent, which had a strong salt tolerance phenotype (Figure 1a). Genotyping for CSSLs was performed with SSR/InDel and SNP markers (Figure S1). To identify genes underlying the natural variations in salt tolerance, the phenotypic datasets were assessed by systemically evaluating the salt tolerance at the seedling stage. High phenotypic variation was detected in six salt tolerance‐related traits in the CSSL populations, with transgressive segregation for each trait (Table S1). Correlation analysis showed that these parameters were positively correlated (Table S2). Fifteen QTLs were identified using software QTL Ici‐Mapping (Table S3). Among them, the QTL *qSSR5.1* near SNP marker S5_27886769 locus contributed to both the seedling survival rate (PVE 8.65%) and seedling salt‐tolerant grade (PVE 8.13%) (Figure 1b; Table S3). Then this locus named as *qST5* was mapped by both SSR/InDel and SNP markers, which contains 13 ORF candidate genes within the 71.4‐kb flanking region on Chr.5 (Figure 1c).

To identify the genetic identity of *qST5*, we integrated the transcriptomic [19] and genomic data between wild rice and 93‐11. We found that 13 genes in the *qST5* locus, except *LOC_Os05g48800*, showed irregular expression levels after salt treatment (Figure S2; Table S4). The *LOC_Os05g48800*, whose expression was significantly repressed in wild rice, was identified in this locus. It encodes a C2H2‐type transcription factor. Sequence analysis revealed that the coding region of this gene had no difference between wild rice and 93‐11, but a 36‐bp InDel natural variation in the promoter harbors two W‐boxes in the wild rice (Figure 1d; Figure S3a). A chromosome segment substitution line CSSL153, which harbors this QTL and showed higher salt tolerance than 93‐11 in the seedling stage, was selected for further study (Figure S3b–d). To identify whether *LOC_Os05g48800* plays a vital role in determining salt tolerance, we designed a CAPS marker in the *LOC_Os05g48800* promoter (Figure S4). A near‐isogenic line (NIL) of *qST5* in BC_2_F_3_ generation was constructed by backcrossing CSSL153 with the recurrent parent 93‐11. The salt tolerance phenotypes of 93‐11 and NIL were investigated. Intriguingly, the NIL showed significantly higher salt tolerance than 93‐11, with a higher survival rate, longer length, and more weight of shoot and root after 150 mM NaCl treatment (Figure 1e–h).

Given the promoter region of *LOC_Os05g48800* had a 36‐bp insertion in wild rice compared with 93‐11 (Figure 1d), we investigated the expression patterns of *LOC_Os05g48800* in both NIL and 93‐11 with salt treatments, the expression of *LOC_Os05g48800* was obviously induced by NaCl treatment in 93‐11, and the transcript level of *LOC_Os05g48800* was significantly repressed in NIL under both normal and salt treatment conditions (Figure 1i). To determine the correlation between *LOC_Os05g48800* and salt tolerance, we crossed 93‐11 with the NIL to produce an F_2_ population, from which more than 2,000 single plants were selected and planted in a paddy field with 150 mM NaCl treatment. We found that the salt‐tolerant plants of F_2_ individuals harbored the wild rice allele, while the salt‐sensitive plants carried the 93‐11 allele (Figure S5). Taken together, we speculated that *LOC_Os05g48800* was the candidate/causal gene and named *ST5*.

### 2.2 *ST5* Negatively Regulates Salt Tolerance

**FIGURE 2:**
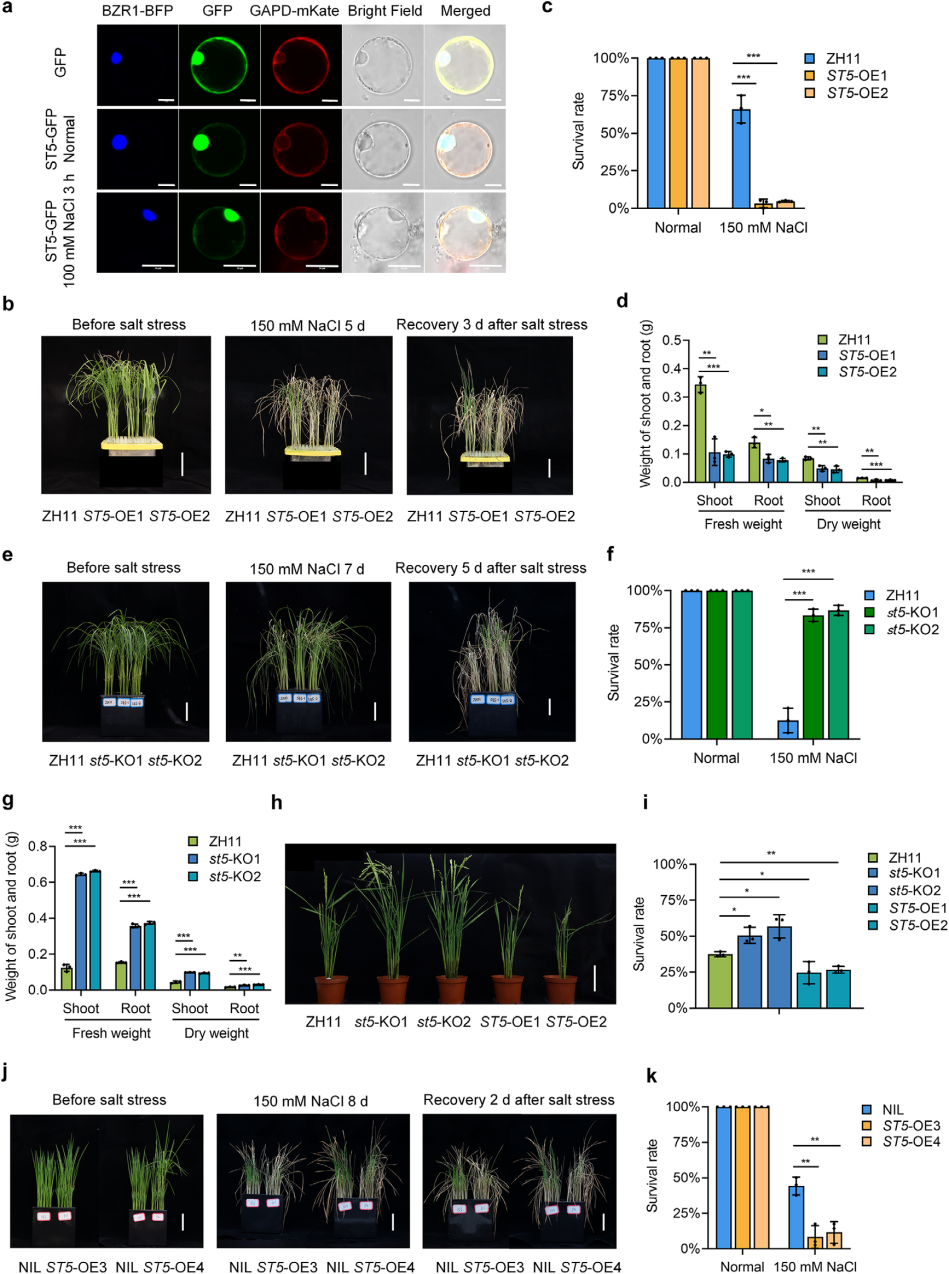
*ST5* negatively regulates salt tolerance in rice. a) Subcellular localization of ST5‐GFP in rice protoplasts. BZR1‐BFP and GAPD‐mKate are markers for nucleus and cytoplasm localization, respectively. Bars = 10 µm. (b‐d) The salt tolerance phenotypes of *ST5* overexpression lines (*ST5‐*OE1 and *ST5‐*OE2) in the ZH11 background (b). Two‐week‐old seedlings were treated with 150 mM NaCl for five days, and then recovered for three days under normal conditions. Survival rate of recovered seedlings (c), and fresh and dry weight of shoot and root of recovered seedlings (d), were investigated. Bar = 5 cm. (e–g) The salt tolerance phenotypes of *ST5* knock‐out lines (*st5‐*KO1 and *st5‐*KO2) in the ZH11 background (e). Two‐week‐old seedlings were treated with 150 mM NaCl for seven days and then recovered for five days under normal conditions. Survival rate of recovered seedlings in (f). Fresh and dry weight of the shoot and root of recovered seedlings are in (g). Bar = 5 cm. (h, i) Phenotypes of *ST5* overexpression and knock‐out lines in paddy field with 51 mM NaCl concentration (h). Bar = 10 cm. Survival rate of rice after two months of transplanting (i). (j,k) The salt tolerance phenotypes of *ST5* overexpression lines (*ST5‐*OE3 and *ST5‐*OE4) in the NIL background (j). Two‐week‐old seedlings were treated with 150 mM NaCl for eight days, and then recovered for two days under normal conditions. Survival rate of recovered seedlings is in (k). Bar = 5 cm. Data are means ± SD (*n* = 3 biological replicates). ^*^, *P* < 0.05; ^**^, *P* < 0.01; ^***^, *P* < 0.001; ns, no significant difference; Two‐tailed Student's *t*‐test.

To analyze the phylogenetic organization of the ST5 protein family, a phylogenetic tree was constructed via the neighbor‐joining method based on the full‐length protein. ST5 was closely clustered with wheat drought‐induced protein TaDi19‐1 (Figure S6), which was reported to be involved in wheat salt stress response [25, 26]. To determine the subcellular localization of ST5, the *35S:ST5‐GFP* constructs and marker were cotransformed into rice protoplasts. The results showed that ST5‐GFP was preferably localized in the nucleus and cytoplasm, and 100 mm NaCl treatment did not alter ST5 subcellular localization (Figure 2a).

To further validate the role of *ST5* in salinity stress tolerance, the overexpression (OE) and knock‐out (KO) lines of *LOC_Os05g48800* were generated in the *japonica* variety ZhongHua11 (ZH11) background (Figure S7a,b). After salt treatments, the *ST5‐*OE lines displayed hypersensitivity to salinity stress with a significant reduction of survival rate compared with wild‐type ZH11 (Figure 2b,c). Meanwhile, the *st5*‐KO lines were more tolerant to salinity stress and had a significantly higher survival rate than ZH11 (Figure 2e,f). A comprehensive assessment of the dry and fresh weight of both root and shoot tissues after salt treatments was conducted (Figure 2d,g). These findings also demonstrated that the *st5*‐KO lines were more tolerant, while the *ST5‐*OE lines displayed less salt tolerance than wild‐type ZH11.

We investigated the yields and agronomic traits of *ST5‐*OE and *st5*‐KO lines under normal conditions. The tiller number has no significant difference among *ST5‐*OE, *st5‐KO*, and wild‐type lines. Plant height of *ST5‐*OE and *st5*‐KO2 lines was significantly decreased compared with ZH11, but there was no difference in the *st5*‐KO1 line. For yield, the *ST5‐*OE lines decreased significantly compared with ZH11, but *the st5*‐KO lines had no difference (Figure S7c–e). Moreover, we also treated the *ST5‐*OE and *st5*‐KO lines with 51 mm NaCl salt concentration in the paddy field, and found that the salt tolerance of the *st5*‐KO lines was significantly higher than that of ZH11, whereas the salt tolerance of *ST5‐*OE lines was significantly lower than that of ZH11 (Figure 2h,i; Figure S7f). These results indicate that *ST5* negatively regulates salt tolerance in rice. Furthermore, the *ST5‐*OE lines in the NIL background showed hypersensitivity to salinity stress with significantly reduced survival rate, compared with NIL (Figure 2j,k). Therefore, we concluded that *ST5* negatively regulated salt tolerance in rice.

### 2.3 Transcription Factor OsWRKY80 Binding to the 36‐bp Insertion in *ST5* ^*W*^ Promoter and Inhibiting Its Expression

**FIGURE 3:**
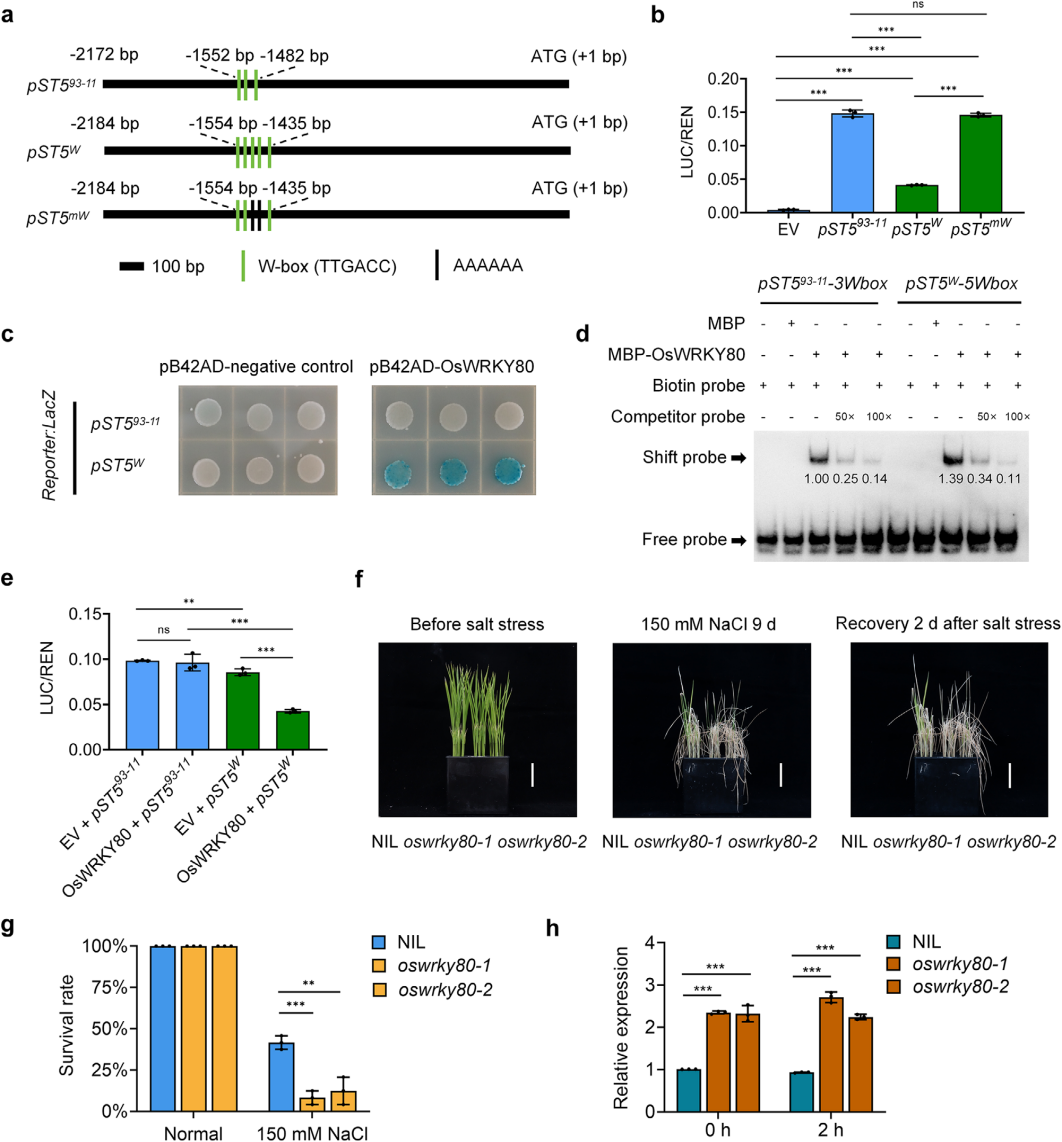
Transcription factor OsWRKY80 binds to the 36‐bp insertion in *ST5^W^* promoter and inhibits its expression. (a) Schematic representation of *ST5^93‐11^*, *ST5^W,^* and *ST5^mW^* promoters. The green lines indicate W‐boxes, the black lines indicate mutated W‐boxes. (b) Transient expression assays in rice protoplasts showing the promoter activities of *pST5^93‐11^*, *pST5^W,^* and *pST5^mW^*. Protoplasts were incubated at 25 °C for 20  h. An empty vector (EV) was included as a control. (c) Y1H assays showing the different binding affinity of OsWRKY80 to *pST5^93‐11^* and *pST5^W^*. (d) EMSA showing the difference in OsWRKY80 binding affinity with five W‐boxes containing the region of *pST5^W^* and three W‐boxes containing the region of *pST5^93‐11^*. The loading amount of MBP or MBP‐OsWRKY80 protein was 200 ng, and the final concentration of probe after annealing was 20 mM. Relative binding intensity was quantified with ImageJ software. (e) Transient dual‐luciferase expression assay of OsWRKY80 in rice protoplasts. Protoplasts were incubated at 25 °C for 20  h. EV was included as a control. (f,g) The salt tolerance phenotypes of *oswrky80‐1* and *oswrky80‐2* knockout lines (f) in the NIL background. Two‐week‐old seedlings were treated with 150 mM NaCl for nine days, and then recovered for two days under normal conditions. Survival rate of recovered seedlings is in (g). Bar = 5 cm. (h) The expression levels of *ST5* in *oswrky80* mutant in the NIL background revealed by qPCR. Data are means ± SD (*n* = 3 biological replicates). ^*^, *P* < 0.05; ^**^, *P* < 0.01; ^***^, *P* < 0.001; ns, no significant difference; Two‐tailed Student's *t*‐test.

A variation of 36‐bp InDel leads to a difference of two W‐boxes in the promoter region, and no other TF motifs were found in this interval. This variation leads to different expression levels of *ST5* between NIL and 93‐11 (Figure 1d,i). We hypothesized that the two W‐box variations affected the promoter activity. To test this, we performed the transient expression assays in rice protoplasts. The results showed that the promoter activity of *ST5* from wild rice (*pST5^W^*) was significantly lower than that of 93‐11 (*pST5^93‐11^*) (Figure 3a,b). Furthermore, we mutated the two W‐boxes in *pST5^W^* and found that the activity of *pST5^mW^* was significantly increased compared with *pST5^W^*, and there was no difference with *pST5^93‐11^* (Figure 3a,b). These results suggest that the 36‐bp InDel that resulted in the difference in the W‐box may lead to different promoter activities between 93‐11 and NIL.

Previous studies demonstrated that the W‐box was the canonical target of a family of WRKY, and the number of W‐boxes has a pivotal effect on the binding ability of WRKY TFs [12]. We therefore hypothesized that WRKY TFs might display differential binding abilities to *pST5^93‐11^* and *pST5^W^*. To test this hypothesis, we cloned three WRKY TFs (OsWRKY13, OsWRKY45, OsWRKY80) that had been shown to be related to salt tolerance of rice [13, 14, 15]. We then detected the interaction of these WRKY TFs with the *ST5* promoters using yeast one‐hybrid assays. The results showed that OsWRKY13 and OsWRKY45 show indiscriminate binding affinity to *pST5^93‐11^* and *pST5^W^*, whereas only OsWRKY80 exhibits differential binding affinity. OsWRKY80 binding to *pST5^W^* and the binding affinity was significantly stronger than *pST5^93‐11^* (Figure 3c; Figure S8). Electrophoretic mobility shift assays (EMSAs) also confirmed that MBP‐OsWRKY80 showed stronger binding ability to *pST5^W^* compared with *pST5^93‐11^* (Figure 3d). Dual luciferase reporter assay showed that OsWRKY80 repressed the activity of *pST5^W^* but not *pST5^93‐11^* in rice protoplasts (Figure 3e).

To functionally determine the involvement of OsWRKY80 in the regulation of *ST5*, we knocked out the *OsWRKY80* gene in the NIL background by CRISPR/cas9 (Figure S9). The *oswrky80‐1* and *oswrky80‐2* lines in the NIL background displayed a salt stress hypersensitive phenotype, with survival rate significantly reduced after salt treatments (Figure 3f,g). We measured the expression levels of *ST5* in the *oswrky80* lines by qPCR. The results showed that the expression levels of *ST5* in *oswrky80* were remarkably up‐regulated compared with those in the NIL (Figure 3h). Taken together, our results demonstrated that OsWRKY80 repressed the expression of *ST5^W^* by binding to W‐boxes.

### 2.4 ST5 Directly Represses the Expression of *OsCPK4* That is Involved in Salt Response and Na^+^/K^+^ Homeostasis

**FIGURE 4:**
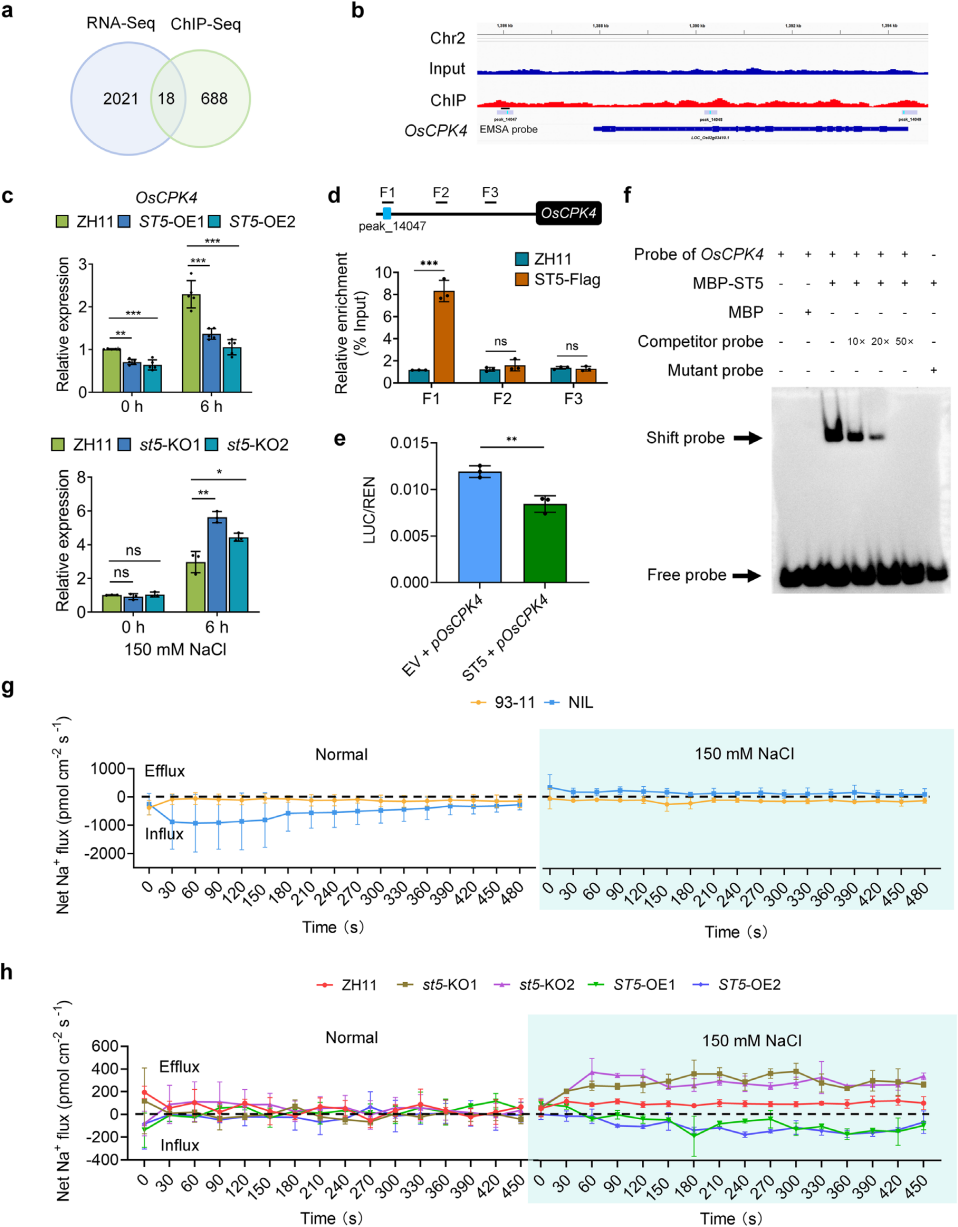
ST5 directly represses the expression of *OsCPK4* and is involved in Na^+^/K^+^ homeostasis. (a) Venn diagram showing shared genes between ST5 target genes in the *ST5* overexpression line compared with WT, and differentially expressed genes (DEGs) in the *ST5* knockout line compared with WT. A significance cutoff of *p* < 0.01, and an absolute fold change of (FC) > 1 were used. (b) Visualization of ST5‐bound peaks on *OsCPK4* in Integrative Genomics Viewer. The genomic regions for inspection are indicated, and sequencing of the input DNA was included as a control. (c) Transcript levels of *OsCPK4* in ZH11, *ST5‐*OE, and *st5*‐KO with or without 150 mM NaCl treatment. (d) Validation of ST5 direct binding sites in the *OsCPK4* promoter by ChIP‐qPCR analysis. The top diagram indicates the fragments used in ChIP‐qPCR. (e) ST5 inhibits the transcription of *OsCPK4* in the dual‐luciferase transient expression assay in rice protoplasts. (f) DNA binding activity of ST5 protein on *OsCPK4* promoter fragment tested by EMSA assays. Probe design position was marked in Figure 4b. (g,h) Net Na^+^ fluxes in 500 µm distance from the root apex of 93‐11, NIL (g), and ZH11, *ST5*‐OE, *st5*‐KO (h) measured using NMT under 150 mM NaCl treatment. Data are means ± SD (*n* = 3 biological replicates). ^*^, *P* < 0.05; ^**^, *P* < 0.01; ^***^, *P* < 0.001; ns, no significant difference; Two‐tailed Student's *t*‐test.

To identify the target genes of ST5 in salt stress response in rice, we performed RNA sequencing (RNA‐seq) analysis using ZH11 and *st5*‐KO seedlings. The analysis revealed 2,039 differentially expressed genes (DEGs) (absolute log_2_ fold change > 1, *p* < 0.01) in *st5*‐KO lines when compared with ZH11. Among them, 1,414 genes were up‐regulated, and 625 were down‐regulated (Figure 4a; Figure S10a,b). Gene ontology analysis indicated that these genes were enriched in biological processes involved in the regulation of response to multiple environmental stresses (Figure S10c). Then, we conducted a chromatin immunoprecipitation sequencing (ChIP‐seq) assay using *ST5*‐OE transgenic seedlings. A total of 2,044 notable peaks were identified, which corresponded to 706 genes (Figure 4a). By performing an overlapping analysis of RNA‐seq and ChIP‐seq data, we identified 18 genes as both directly bound genes and DEGs (Figure 4a; Figure S10d), which were thus considered as the direct targets of ST5. According to the rice database (https://ricedata.cn/), seven of them have been functionally characterized (Table S5). Among them, *OsCPK4* was a promising target regulating salt tolerance in rice [27, 28, 29]. In addition, further inspection using the Integrated Genome Viewer indicated that the binding peaks on *OsCPK4* were highly specific (Figure 4b). To validate this finding, we performed qPCR to investigate the expression patterns of *OsCPK4* in *ST5*‐OE and *st5*‐KO lines. The results showed that the expression of *OsCPK4* was drastically down‐regulated in the *ST5*‐OE seedlings and significantly up‐regulated in the *st5*‐KO with NaCl treatments (Figure 4c). ChIP‐qPCR assays demonstrated the significant enrichment of ST5 associated with the F1 region of the *OsCPK4* promoter (Figure 4d). Rice protoplast dual luciferase reporter assay showed that ST5 inhibited *OsCPK4* transcription (Figure 4e). EMSA assays demonstrated that ST5 could bind to the promoter of *OsCPK4* (Figure 4f). These results indicate that ST5 directly targets the *OsCPK4* gene to repress its transcription.

As *OsCPK4* was correlated significantly with Na^+^/K^+^ homeostasis, we measured the Na^+^ and K^+^ fluxes in 93‐11, NIL, and ZH11, *st5*‐KO, and *ST5*‐OE lines using Non‐invasive Micro‐test Technology (NMT). The NIL showed higher Na^+^ efflux and K^+^ influx under salt stress compared with 93‐11 (Figure 4g and Figure S11a). Similarly, *st5*‐KO showed higher Na^+^ efflux under salt stress compared with ZH11, whereas *ST5*‐OE exhibited lower Na^+^ efflux than ZH11 and *st5*‐KO (Figure 4h). There was no difference in K^+^ flux among ZH11, *ST5*‐OE, and *st5*‐KO lines (Figure S11b). These results demonstrated that *ST5* regulated Na^+^/K^+^ homeostasis.

### 2.5 The Superior *ST5^W^* Allele Originates From Wild Rice *O. rufipogon*

**FIGURE 5:**
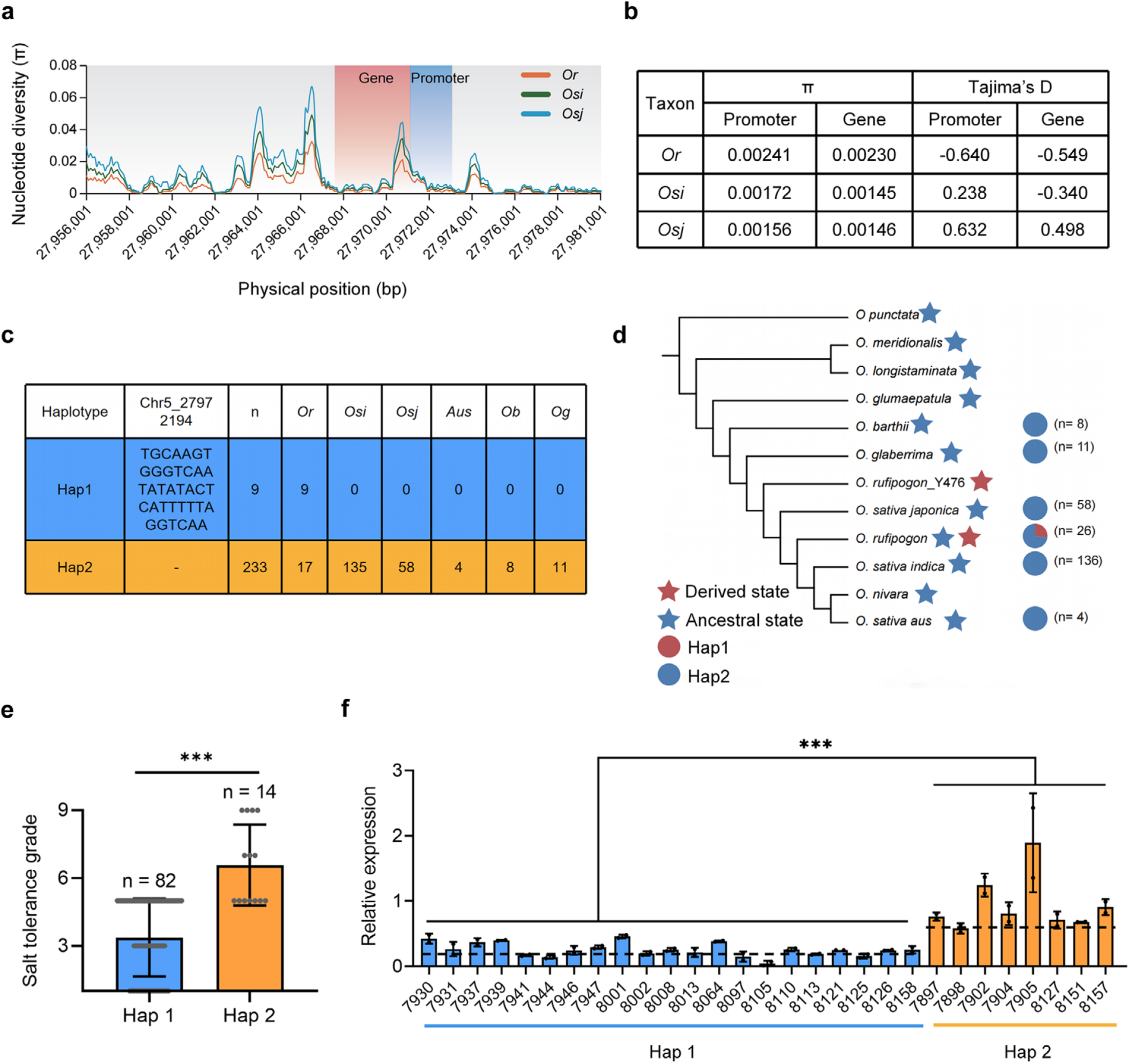
Haplotype, evolutionary origin, and relative expression analysis of *ST5* in rice germplasm resources. (a) Nucleotide diversity of *ST5* and flanking regions in cultivated rice and wild rice populations. The positions of the *ST5* gene and promoter region were marked with red and blue colors, respectively. (b) Nucleotide diversity (π) and Tajima's D tests were performed with 26 *O. rufipogon*, 136 *O. sativa indica*, and 58 *O. sativa japonica* accessions. (c) Haplotype analysis of the 36‐bp InDel of *ST5* based on a Mini‐Core of rice accessions. n, number of accessions. *Or*, *Osi*, *Osj*, *Aus*, *Ob*, and *Og* refer to *O. rufipogon*, *O. sativa indica*, *O. sativa japonica*, *O. sativa aus*, *O. barthii*, and *O. glaberrima*, respectively. (d) Phylogeny analysis of the AA wild rice accessions based on the promoter sequence of *ST5*. A wild rice specie of the BB genome, *O. punctata*, was used as an outgroup. (e) Salt tolerance grade for two haplotypes in *O. rufipogon*. *n* is the number of samples. (f) Relative expression of *ST5* in *O. rufipogon* accessions in response to salt stress. The relative expression level of *ST5* at 6 h after salt stress (200 mM NaCl) was quantified by qPCR. Data are means ± SD (*n* = 2 biological replicates). ^***^, *P* < 0.001; Two‐tailed Student's *t*‐test.

To ascertain whether *ST5* underwent selection during rice evolution, we calculated nucleotide diversity (π) across its promoter and coding regions using high‐quality chromosome‐level assembled and annotated genomes from the super pan‐genomic landscape [18]. Similar diversity was observed among *O. rufipogon*, *O. indica*, and *O. japonica* accessions (Figure 5a,b), and no significant Tajima's D value (*p* > 0.10) was detected in *O. indica* and *O. japonica* accessions (Figure 5b). These findings suggested that *ST5* did not exhibit significant selection signals.

We further investigated the frequency of the 36‐bp insertion in the super pan‐genomic dataset and found that it was present specifically in the wild rice *O. rufipogon*, but absent in the Asian cultivated rice or African rice accessions. The accessions with the 36‐bp insertion were named as Hap1, and the other accessions without this insertion were named Hap2 (Figure 5c). We expanded the rice population to a large‐scale gene pool of 10 548 rice accessions [30], and found that the 36‐bp W‐boxes insertion is only found in approximately 10% of *O. rufipogon*, while it is absent in all the Asian cultivated rice accessions (Figure S12a). We further traced the evolutionary trajectory of the 36‐bp W‐boxes insertion across the AA‐genome species and the other *Oryza* genome types in the *Oryza* genus by constructing a phylogenetic tree. The result revealed that the 36‐bp W‐boxes insertion is a derived status of this locus and is only detected in *O. rufipogon*, and absent in all the other wild and cultivated rice species (Figure 5d; Figure S12b), indicating a *de novo* origin of this variant and a potential non‐selection for it in the rice domestication.

We collected 530 accessions of *O. rufipogon* all over China, which represented most genetic diversity as the core germplasm of Chinese common wild rice (Figure S13a). The salt tolerance grade of this population was evaluated. The genotypes of *ST5* were detected using CAPS molecular markers; most Hap 1 have higher salt tolerance than Hap 2 (Figure 5e; Figure S13b). Moreover, the expression levels of *ST5* in Hap 1 individuals were much lower than those of Hap 2 (Figure 5f). Taken together, these findings suggest that the *ST5^W^* allele is a rare allele that has not been selected in rice domestication and has great application potential in improving salt tolerance of cultivated rice.

### 2.6 *ST5^W^* Allele Enhances Salt Tolerance in Multiple Cultivated Rice Backgrounds

**FIGURE 6:**
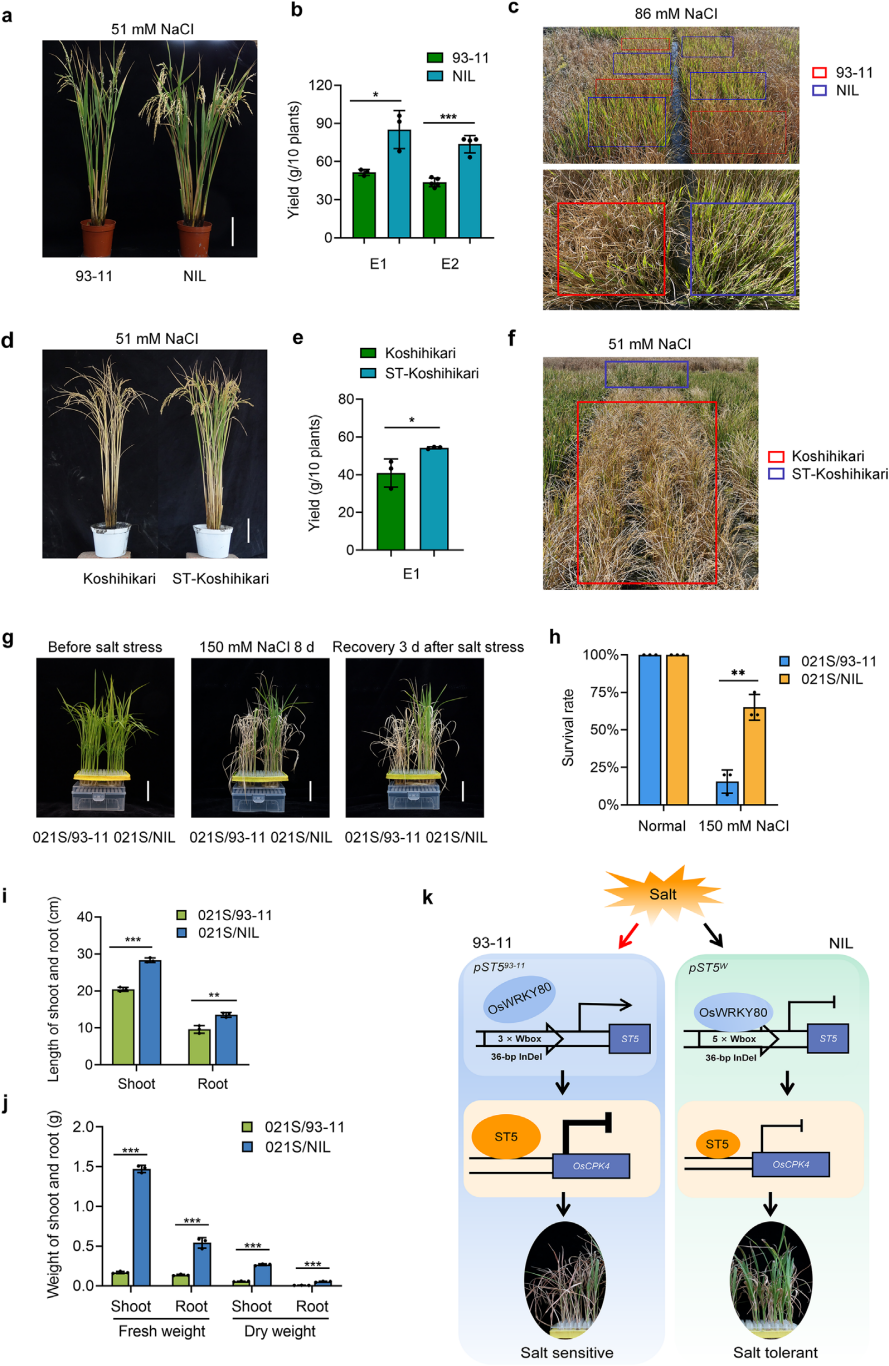
The wild rice allele *ST5^W^* enhances salt tolerance in multiple cultivated rice backgrounds. (a) Phenotypes of 93‐11 and the NIL individuals grown under saline field conditions. The salt contents in soils before ploughing are shown in brackets. Bar = 10 cm. (b) Statistics of grain yields per ten plants of 93‐11 and NIL in the paddy field with 51 mM NaCl salinity in two environments. E1 is Dongying city in summer, and E2 is Sanya city in winter. Data are means ± SD (*n* ≥ 3 biological replicates). (c) Picture of 93‐11 and NIL plants in a paddy field with 86 mM NaCl salinity in E1. The red box represents 93‐11, and the blue box represents the NIL. (d) Phenotypes of Koshihikari and ST‐Koshihikari individuals grown under saline field conditions. The salt contents in soils before ploughing are shown in brackets. Bar = 10 cm. (e) Statistics of grain yields per ten plants of Koshihikari and ST‐Koshihikari under 51 mM NaCl saline field in E1. Data are means ± SD (*n* = 3 biological replicates). (f) Picture of Koshihikari and ST‐Koshihikari plants in a paddy field with 51 mM NaCl salinity in E1. The red and blue boxes represent Koshihikari and ST‐Koshihikari, respectively. (g‐j) Salt tolerance phenotype of hybrid rice 021S/93‐11 and 021S/NIL (g). The survival rates (h), length of shoot and root (i), and fresh and dry weight of shoot and root (j) were determined after a three‐day recovery period. Bars = 5 cm. Data are means ± SD (two‐tailed Student's *t*‐test; *n* = 3 biological replicates). ^*^, *P* < 0.05; ^**^, *P* < 0.01; ^***^, *P* < 0.001. (k) Proposed model of *ST5* regulated salt tolerance in rice. In rice, a 36‐bp natural variation in the promoter of *ST5* results in two distinct promoter types, *pST5^93‐11^* and *pST5^W^*. OsWRKY80 directly binds to W‐boxes residing within the 36‐bp sequence of the *pST5^W^*, leading to suppressed expression of *ST5*. Decreased expression of *ST5* reduces inhibition to the *OsCPK4*, resulting in an increasing of the expression level of *OsCPK4*, and confers salt tolerance in rice through improved plant Na^+^/K^+^ homeostasis (right). In contrast, OsWRKY80 does not bind to *pST5^93‐11^*, leading to relatively higher expression of *ST5* and lower salt tolerance (left).

Since the cultivated rice does not have the *ST5^W^* allele, we further explored the potential for application of the *ST5^W^* allele to increase salt tolerance in *indica* and *japonica*. Previously, we constructed the NIL by backcrossing the *indica* cultivar 93‐11 with wild rice. As we expected, the survival rate of the NIL was significantly higher than that of the 93‐11 plants at the seedling stage (Figure 1e,f). Then, we conducted field experiments. Under normal conditions, the yield traits had no significant difference between the NIL and 93‐11 (Figure S14a–c). We transplanted one‐month‐old 93‐11 and NIL seedlings into fields with a salt content of either 51 or 86 mM NaCl. We found that the yield per ten plants of the NIL was significantly higher than that of 93‐11 under 51 mM NaCl salt conditions in two environments (Figure 6a,b). And the NIL could survive under 86 mM NaCl salt stress, but 93‐11 did not (Figure 6c).

To verify whether or to what extent the *ST5^W^* allele can make a bona fide contribution to salt tolerance, we constructed a BC_4_F_3_ population by backcrossing the salt‐sensitive *japonica* cultivar Kaoshihikari (as recurrent parent) with the *indica* NIL (as donor parent) using molecular marker‐assisted selection (Figure S14d). A line harboring the *ST5^W^* allele in the Kaoshihikari background was developed and named ‘ST‐Kaoshihikari’. The ST‐Kaoshihikari showed no significant difference in yield and other agronomic traits compared with Kaoshihikari under normal conditions (Figure S14e–g). Evaluations of salt tolerance under 51 mM NaCl salt stress confirmed that the ST‐Kaoshihikari was more tolerant than Kaoshihikari, with a significantly improved yield under salt stress (Figure 6d–f). Meanwhile, considering that 93‐11 is a hybrid rice restorer line, the *ST5^W^* allele and *ST5^93‐11^* allele were introduced into the photosensitive male sterile line 021S by crossing the NIL and 93‐11 with the 021S line, respectively. The 021S/NIL and 021S/93‐11 hybrids were treated with salt stress at the seedling stage. The 021S/NIL hybrids showed significantly enhanced survival rate and salt tolerance compared with 021S/93‐11 hybrids (Figure 6g–j). Taken together, the rare *ST5^W^* allele offers a viable target for improving salt tolerance in cultivated rice.

## 3 Discussion

Wild rice harbors abundant beneficial alleles that have been lost during the breeding of modern varieties, which represent a valuable resource for breeding programs aimed at producing cultivars with better resistance to biotic and abiotic stresses, as well as for research aimed at understanding the mechanisms underlying stress resistance in plants. In this study, we identified a rare wild rice allele *ST5^W^* that encodes a C2H2‐type TF, using a CSSL population. Many stress‐tolerant genes were tightly linked with inferior yield‐related characters in wild rice, i.e., linkage drag, which is the reason that most of the elite salt tolerance genes were not selected during rice domestication. Our CSSL population has greatly facilitated the discovery of unknown useful genes in wild rice by minimizing linkage drag [19]. By testing salt tolerance in the NIL, the F_2_ segregating population of NIL/93‐11, *st5*‐KO, and *ST5*‐OE lines, we confirmed that *LOC_Os05g48800* is the causal gene of *qST5* (Figures 1 and 2). The CAPS marker we designed, linked with *ST5^W^*/*LOC_Os05g48800* provided a tool for marker‐assisted selection breeding.

We proposed a possible working model for the *ST5, which* involved a salt tolerance regulation network and showed that the rare allele played an essential role in salt tolerance (Figure 6k). A 36‐bp insertion was found in the wild rice allele *ST5^W^* promoter region, which contains two W‐box motifs. We identified that OsWRKY80 specifically bound to the 36‐bp insertion region and reduced the expression of *ST5^W^* (Figure 3). ST5 directly represses the expression of *OsCPK4* and regulates Na^+^/K^+^ homeostasis (Figure 4). The *OsWRKY80*‐*ST5*‐*OsCPK4* module provides new insights into the regulatory network underlying rice salt tolerance.

Moreover, we identify *ST5^W^* as a rare elite allele associated with salt tolerance that is uniquely present in *O. rufipogon*. Among the AA genome species, the *ST5^W^* allele, which is characterized by a 36‐bp W‐box insertion, was detected in a subset of *O. rufipogon* accessions with a prevalence of only 10% (Figure 5; Figure S12). This observation indicates a narrow natural distribution for this unique allele. Phylogenetic analysis revealed that the 36‐bp W‐boxes insertion was uniquely present in *O. rufipogon* and was absent from all the other wild and cultivated rice species (Figure 5). Our findings suggest that this allele originated *de novo* within *O. rufipogon* but was subsequently eliminated during the domestication process.

*The ST5^W^* allele showed strong salt tolerance in multiple genetic backgrounds, suggesting that *ST5^W^* could be a suitable genetic target for rice salt tolerance breeding. 93‐11 is an elite *indica* variety with high biotic and abiotic stress resistance; it is difficult to improve salt tolerance by changing a single gene in the 93‐11 genetic background. However, we showed that the NIL displayed enhanced salt tolerance in a saline paddy field at both the seedling and adult stages. The NIL can even survive in a field with 86 mM NaCl salt concentration. Our field trials, which were carried out in multiple saline paddy fields, showed that the introduction of *ST5^W^* from wild rice into cultivars resulted in around 30–50% yield improvement in *indica* and *japonica* backgrounds (Figure 6). The unique presence of this allele in *O. rufipogon*, while being absent in agricultural production, underscores its potential as an underexploited genetic resource. This genetic resource could significantly benefit modern rice breeding programs aimed at enhancing stress tolerance, paving the way for its incorporation into elite cultivars and contributing to the development of stress‐resilient rice varieties.

## 4 Experimental Section

### 4.1 Plant Materials and Growth Conditions

The CSSLs were generated in our previous studies [19, 24]. We backcrossed CSSL153 with the recurrent parent 93‐11 to construct the NIL. All wild rice accessions and rice varieties in this study were conserved in our laboratory. Unless specifically described, all the rice seedlings were growing under a 16 h light and 8 h dark photoperiod at 28 ± 2°C in the growth chamber. For field trial, after ten days of transplanting, rice grown in paddy field was irrigated with NaCl solution with 51 or 86 mM concentration in Dongying city, Shandong province, China (E1, N36°55, E118°07′) in summer season (1st May to 5th Oct), and Sanya city, Hainan province, China (E2, N18°9′, E108°56′) in winter season (1st Nov to 20th Apr), respectively. Survival rates were calculated after two months of transplanting. All agronomic and yield traits measurements were based on at least 20 individuals, which were randomly selected.

For identification of *ST5* overexpression homozygous lines, genomic DNA was isolated from leaves of T2 transgenic and wild‐type plants using the CTAB method. The integration of the transgene was confirmed by polymerase chain reaction (PCR) using gene‐specific primers listed in Table S6. The PCR products were separated by electrophoresis on a 1.0% agarose gel and visualized under UV light. To characterize *st5* CRISPR/Cas9 knock‐out lines, genomic DNA from T2 plants was used as a template for PCR amplification. The gene‐specific primers used are shown in Table S6. Multiple independent plants per line (minimum of 6) were sequenced using Sanger sequencing. The sequences were aligned to the wild‐type reference sequence using SnapGene to identify insertions, deletions, or substitutions.

### 4.2 Phenotype Identification and QTL Mapping

Rice seeds of CSSLs were immersed in sterile water at 37°C for three days. Germinated seeds were then placed into a bottomless 96‐well plate that contained liquid Yoshida's nutrient solution. Salt tolerance indexes were investigated at germination and seedling stages, based on established evaluation criteria for rice germplasm [31]. QTLs for each trait were identified with QTL IciMapping 4.1 [32]. The RSETP‐LRT‐ADD map ping method was applied with a logarithm of odds (LOD) threshold of 2.5.

For the seedling stage of NaCl treatment, two‐week‐old seedlings were transferred to Yoshida's nutrient solution containing NaCl for growing with the number of days indicated in the figures. Subsequently, they were moved to normal Yoshida's nutrient solution for recovery. After an alternative duration as noted, the survival rate was calculated according to the percentage of viable seedlings. Other physiological indexes were also measured. All experiments were repeated three times independently. For the investigation of salt tolerance in wild rice germplasm, individuals with similar growth vigor from each accession were potted in the same soil. After growing for 10 days, wild rice accessions were treated with 200 mM NaCl for seven days, and the salt tolerance grade was investigated.

### 4.3 Development of CAPS Marker

*ST5* promoter‐specific primers (Table S6) were designed, and amplification products were purified using FastPure Gel DNA Extraction Mini Kit (Vazyme) after being verified by agarose electrophoresis. The purified PCR products were digested by *Sca* I (NEB) in a total volume of 50 µL according to the manufacturer's instructions. The digested products were observed by electrophoresis using 2% agarose gel, then photo‐documented using the Gel Imaging Documentation System (Bio‐Rad).

### 4.4 RNA Isolation and qPCR Analysis

Total RNA was isolated using the RNA Easy Fast Plant Tissue Kit (TIANGEN) and reverse‐transcribed into the first‐strand cDNA with a PrimeScript RT Reagent Kit (Takara). Real‐time qPCR experiments were performed using a SuperReal PreMix Plus (SYBR Green) (TIANGEN) on an Applied Biosystems 7500 Real‐Time PCR system following the manufacturer's instructions. All the qPCR analyses were performed for at least three independent biological replicates. Rice *ACTIN* was used as an internal control for all qPCR analyses. Primers for qPCR are listed in Table S6.

### 4.5 Subcellular Localization

To analyze the subcellular localization of ST5 in rice protoplasts, the coding sequences of *ST5* were amplified from cDNA of 93‐11 using specific primers listed in Table S6, and then cloned into *pAN580* (which had been linearized with *Spe* I and *Xba* I). Rice protoplast preparations and transfections were performed as previously described [33]. The protoplasts were incubated at 25°C in the dark for 18 h after transformation. After overnight incubation, 100 mM NaCl was added 3 h before observation. The transfected protoplasts were observed using a fluorescence microscope (Nikon‐AX).

### 4.6 Promoter Activity Transient Expression Assays

The transient expression assays were performed in rice protoplasts. The 2‐kb promoter sequences of *ST5^93‐11^* and *ST5^W^* were amplified from genomic DNA, *ST5^mW^* was synthesized using the standard gene synthesis method of Sangon Biotechnology, and fused with the luciferase reporter gene (LUC) into the plant binary vector *pGreen II‐0800* to generate the reporter constructs *pST5^93‐11^:LUC*, *pST5^W^:LUC* and *pST5^mW^:LUC*. The reporter constructs were transfected into rice protoplasts and incubated at 25°C in the dark for 20 h. The protoplasts were collected by centrifugation at 500 g for 3 min, followed by measurement of LUC and REN activities. The luciferase signal was measured via the Dual‐Luciferase Reporter Assay system (Promega) on a VICTOR Nivo luminometer (PerkinElmer). Relative LUC activity was calculated by normalizing LUC activity to REN activity. Transfection experiments were performed three times.

### 4.7 Dual‐Luciferase Transcriptional Activity Assay

To construct plasmids *ST5^93‐11^pro:LUC*, *ST5^W^pro:LUC*, and *OsCPK4pro:LUC* plasmids, we amplified the promoter sequences from 93‐11 and NIL, and cloned them into the *pGreen II‐0800* vector. The resulting vectors were used as reporters. The full‐length cDNA of *OsWRKY80* and *ST5* were cloned into the *pGreenII 62‐SK* vector to construct *35S:OsWRKY80* and *35S:ST5*, which were used as the effectors. Reporter (*ST5^93‐11^pro:LUC*, *ST5^W^pro:LUC* or *OsCPK4pro:LUC*) and effector (*62‐SK EV*, *35S:OsWRKY80* or *35S:ST5*) were cotransfected into rice protoplasts and incubated at 25°C in the dark for 20 h. The protoplasts were collected by centrifugation at 500 g for 3 min, followed by measurement of LUC and REN activities. The luciferase signal was measured via the Dual‐Luciferase Reporter Assay system (Promega) on a VICTOR Nivo luminometer (PerkinElmer). Relative LUC activity was calculated by normalizing LUC activity to REN activity. Transfection experiments were performed three times.

### 4.8 Yeast One‐Hybrid Assays

The promoter around 36‐bp InDel sequences of *pST5^93‐11^* and *pST5^W^* were amplified from 93‐11 and the NIL, respectively, and cloned into *pLacZ 2ui*. The full‐length cDNAs of the *OsWRKY80* was inserted into the yeast vector *pB42AD*. Meanwhile, *pB42AD* was used as a negative control. To detect protein‐DNA interactions, the combinatorial plasmids were co‐transformed into the yeast strain EGY48 and cultured on a plate containing SD/‐Trp/‐Ura medium for three days at 30°C. Then, yeast cells were transferred and grown on reporter plates containing SD/‐Trp/‐Ura/Gal/Raf/X‐gal medium for three days at 30°C for blue color development.

### 4.9 EMSA

The full‐length cDNA of *OsWRKY80* and *ST5* was cloned into the *pMAL‐c2x* vector. The recombinant MBP‐OsWRKY80, MBP‐*ST5*, and MBP proteins were expressed in *Escherichia coli* BL21 (DE3) cells, and purified following the manufacturer's instructions. Biotin‐labeled and unlabeled probes, synthesized as forward and reverse strands, were added to the reactions. EMSA was performed using a chemiluminescent EMSA kit (Thermos Fisher Scientific, Cat. No. 20148) as described [34]. The *pST5^93‐11^‐3Wbox* probe was derived from a DNA fragment from −1,556 to −1,481 bp of the 93‐11 promoter, and *pST5^W^‐5Wbox* probe was from the −1,595 to −1,481 bp of the wild rice Y476 promoter. The probe sequences are listed in Table S6.

### 4.10 RNA‐Seq

The transcriptomes were analyzed using three biological replicates of RNA samples extracted from the leaves of two‐week‐old seedlings of wild‐type ZH11 and *st5‐*KO mutant. The total RNAs were extracted using Trizol reagent. For each sample, three individual plant leaves were harvested. The RNA samples described above were sequenced using the Illumina Nova Seq 6000 platform. The reads generated were first processed to remove the adapter sequences and low‐quality (Q score < 10) reads. Then reads were mapped to the Nipponbare reference genome (MSU v7) using HISAT2 with default parameters. Differential expression analysis was performed using DESeq2. Differentially expressed genes were identified as those with a *p‐*value of differential expression above the threshold (*p* < 0.01, absolute log_2_ fold change > 1). GO enrichment analysis was performed for the accessions with DEGs via ClusterProfiler.

### 4.11 ChIP‐Seq

Approximately 10 g of leaves of two‐week‐old seedlings of wild type and *ST5*‐OE were used for ChIP‐seq for one biological replicate. Tissue fixation, nuclei extraction, and chromatin immunoprecipitation were performed using anti‐Flag (Sigma, Cat. No. F1804) antibody. The ChIP‐seq DNA libraries were sequenced using the Illumina Nova Seq 6000 platform. After quality control, bowtie2 software was used to align the clean reads against the Nipponbare reference genome (MSU v7). MACS2 software was used for peak calling on a genome‐wide basis, and the threshold for screening significant peaks was *p* < 0.05. Significant peaks were assigned to the nearest gene.

### 4.12 ChIP‐QPCR Analysis

ChIP‐qPCR using the *35S:ST5‐Flag* transgenic plants was performed as described with minor modifications [35]. Anti‐Flag antibody (Sigma, F1804) was used for detection. qPCR reactions were performed in triplicate for each sample, and expression levels were normalized to the input sample for enrichment detection. The ChIP‐qPCR primers are listed in Table S6.

### 4.13 Measurements of the Na^+^ and K^+^ Fluxes

The Na^+^ and K^+^ fluxes were measured using Non‐invasive Micro‐test Technology (NMT) [15]. Seedlings were fixed under Na^+^ and K^+^ ion‐selective microelectrodes in the measuring solution. Net Na^+^ and K^+^ efflux scan at different distances from the root apex was used to confirm the maximum Na^+^ and K^+^ flux region (500 µm distance from the root apex). Na^+^ and K^+^ fluxes were measured for more than 7 min. Approximately 5–10 biological replicates were used.

### 4.14 Nucleotide Diversity, Haplotype, and Evolutionary Analysis of *ST5*

Variant data from the super pan‐genome dataset [18] were filtered using VCFtools v0.1.16 [36] using parameters of allele frequency ≥ 0.05 and maximum missing rate ≤ 0.1. Nucleotide diversity (π) was calculated within 500‐bp windows with 100‐bp steps across the 20‐kb flanking regions, the 2‐kb promoter region, and the coding region of *ST5* using VCFtools v0.1.16. The mean values of the flanking regions were compared. Tajima's D was calculated using DnaSP v6 [37] after manually curating the alignment of the 2‐kb promoter and gene region sequences of *ST5* from the *O. rufipogon*, *O. sativa indica*, and *O. sativa japonica* accessions.

To investigate the frequency of the key variant at this locus (36‐bp W‐boxes insertion), the sequence of the 2‐kb promoter of *ST5* was aligned to the high‐quality assembled pan‐genome [18], the AA‐genome [38], and other *Oryza* genomes [39] using BLASTN v2.16.0+ with default parameters [40]. Subsequently, aligned sequences were extracted using bedtools v2.30.0 [41]. Extracted sequences from the super pan‐genome and the AA‐genome, together with the 2‐kb promoter of *ST5*, were aligned with MAFFT using default settings in Jalview v2.11.4.1 [42]. The frequency of the 36‐bp W‐boxes insertion was then determined based on the alignment. The presence of the 36‐bp W‐boxes insertion in other *Oryza* genomes was also determined based on the alignment of the 2‐kb promoter and coding sequence of *ST5* extracted from their genomes [39]. This frequency across the 10,548 rice accessions [30] was calculated using the variant dataset. Variants in the promoter region of *ST5* were identified using VCFtools v0.1.13, and their frequencies were calculated. Samples with no sequence data or heterozygous genotypes at this locus in the variant dataset were excluded from the statistical analysis. To track the evolutionary trajectory of the 36‐bp W‐boxes insertion across the AA‐genome species of the *Oryza* genus, Neighbor‐Joining analysis was performed on alignments of *ST5* sequences from the AA‐genome species and Y476 using default settings in Jalview v2.11.4.1.

### 4.15 Accession Numbers

Sequence data from this article can be found in the Rice Genome Annotation Project databases (http://rice.plantbiology.msu.edu/) under the following accession numbers: *ST5* (*LOC_Os05g48800*), *OsWRKY80* (*LOC_Os09g30400*), *OsCPK4* (*LOC_Os02g03410*).

### 4.16 Statistical Analysis

Statistical analyses were conducted using Microsoft Excel, where mean values and standard deviations (SD) were calculated. The significance of differences between the two groups was determined using the Student's *t*‐test. *A p*‐value of < 0.05 was considered statistically significant.

## Author Contributions

W.Q., J.S., and L.S. designed the research. Q.Q. and Q.Y. supervised the research project. M.X. performed most of the experiments, and M.X. and W.Q. wrote the manuscript. J.H. conducted QTL mapping and data analyses. Q.Y., H.Q. and X.Z. conducted haplotype and evolutionary analysis. Z.Y., Y.Y., M.Z., and Y.N. contributed to the transgenic experiments and functional analysis. R.X. contributed to the seedling salt tolerance investigation. Q.D., L.G., C.Z., and X.X. contributed to paddy field salt stress experiments. S.W., Y.L., Z.C., L.L., and H.P. participated in the field investigation. W.C., Q.Z., and L.Z. contributed to field management and logistic works. All authors read and approved the final manuscript.

## Conflicts of Interest

The authors declare no conflict of interest.

## Supporting information




**Supporting File 1**: advs75600‐sup‐0001‐FigureS1‐S14.docx.

**Supporting File 2**: advs75600‐sup‐0002‐TableS1‐S6.docx.

**Supporting File 3**: advs75600‐sup‐0003‐DataFile.xlsx.

## Data Availability

The data that support the findings of this study are available in the supplementary material of this article.
