# Supplementary material for: A Rare Allele of ST5 From Wild Rice Enhances Salt Tolerance in Rice
Source: Adv Sci (Weinh). 2026 May 28;13(43):e16159. doi: 10.1002/advs.202516159 (PMC13336088; doi:10.1002/advs.202516159)
Supplement: Supplementary file 1 — Supporting File 1: advs75600‐sup‐0001‐FigureS1‐S14.docx. [file ADVS-13-e16159-s002.docx]

**A rare allele of *ST5* from wild rice enhances rice salt tolerance**

Meng Xing^1, 2^, Jingfen Huang^1, 2^, Qiaoling Yuan^3^, Ziyi Yang^1^, Yanyan Wang^1^, Mingchao Zhao^4^, Yamin Nie^1^, Rui Xu^1^, Hongge Qian^3^, Wenxi Chen^1,2^, Qiaoling Zhang^2, 5^, Qi Du^6^，Leiyue Geng^6^，Yapeng Li ^4^, Ziyi Chen^1^, Shizhuang Wang^1^, Like Lou^2,7^, Haiyuan Peng^1,2^, Chongke Zheng^8^, Xianzhi Xie^8^, Xiaoming Zheng^1^, Lifang Zhang^1^, Lianguang Shang^3*^, Jiaqiang Sun^1*^, Qian Qian^1,3,7,9*^, Qingwen Yang^1,2*^, Weihua Qiao^1,2*^


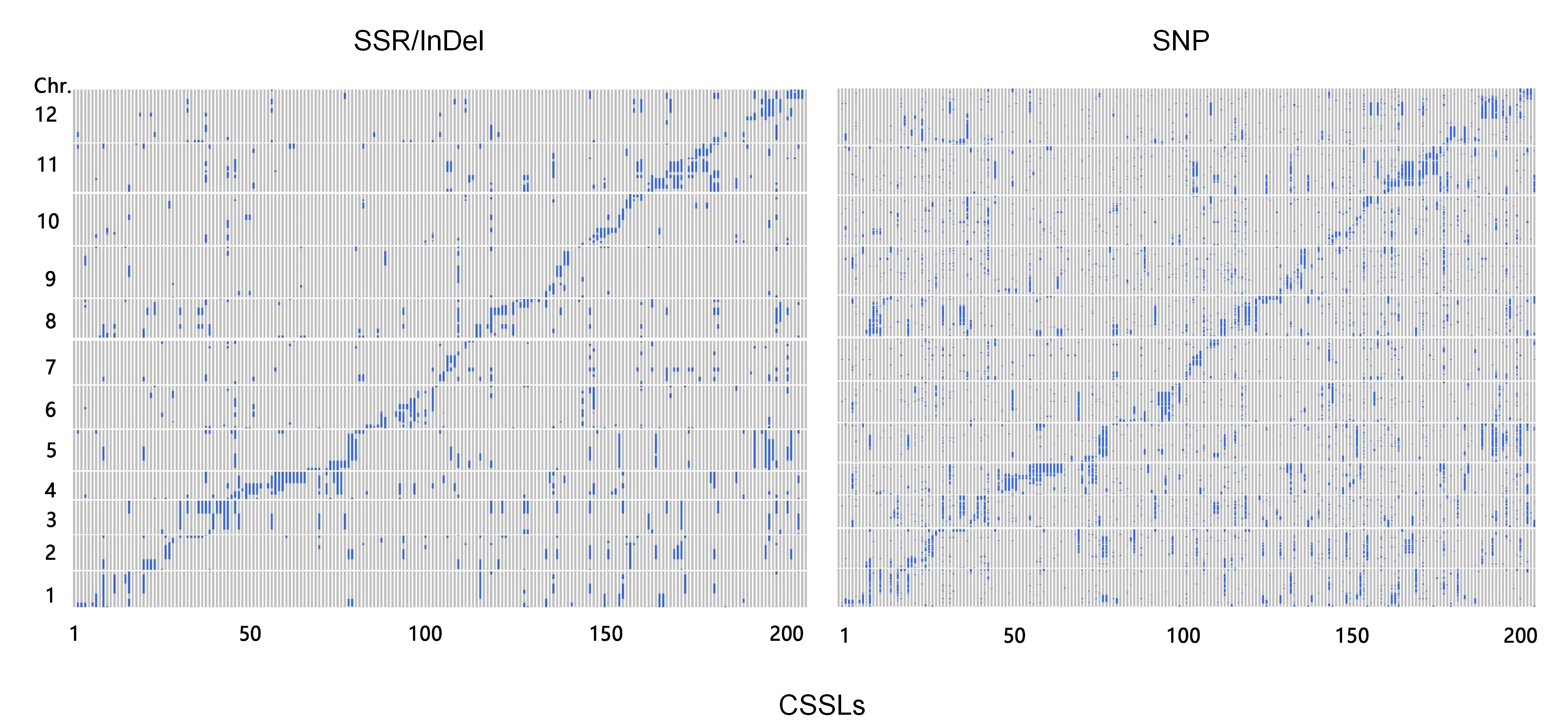


**Figure S1. Graphics of genotypes of CSSLs.**

Regions with a gray background represent homozygous segments from 93-11, blue regions indicate homozygous segments from the donor parent, wild rice Y476. The SNP markers were from genomic sequencing of all CSSLs. The x-axis represents the CSSLs material number, the y-axis represents the 12 chromosomes.


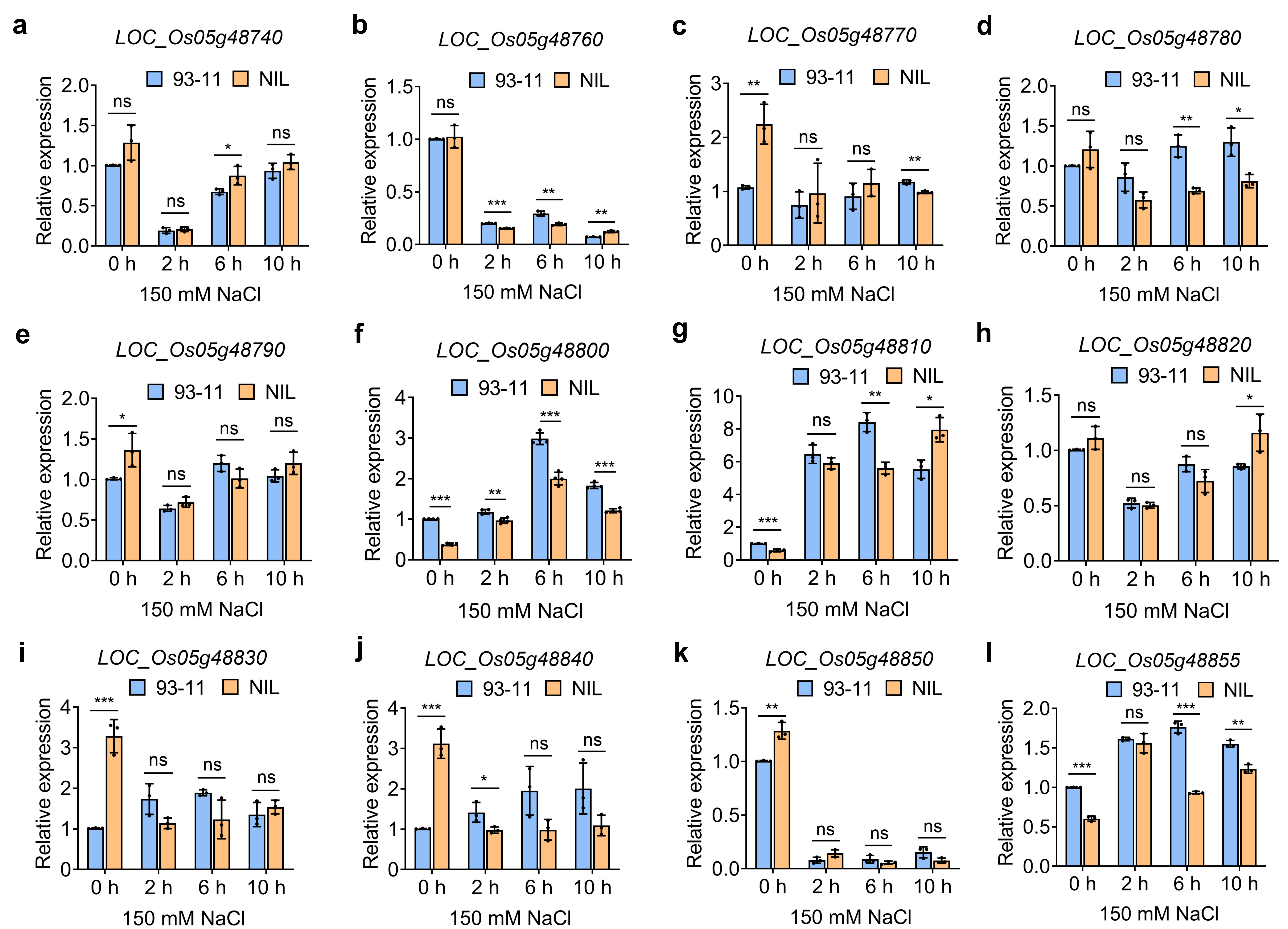
**Figure S2. Analysis of expression patterns of** **13 genes in *qST5* locus after salt treatment.**

The relative expression of 13 genes in *qST5* locus in 93-11 and the NIL with different treatment times in 150 mM NaCl solution. Among them, the expression levels of *LOC_Os05g48750* could not be detected. Data are means ± SD (*n* = 3 biological replicates). *, *P* < 0.05; **, *P* < 0.01; ***, *P* < 0.001; ns, no significant difference. Two-tailed Student’s *t*-test.


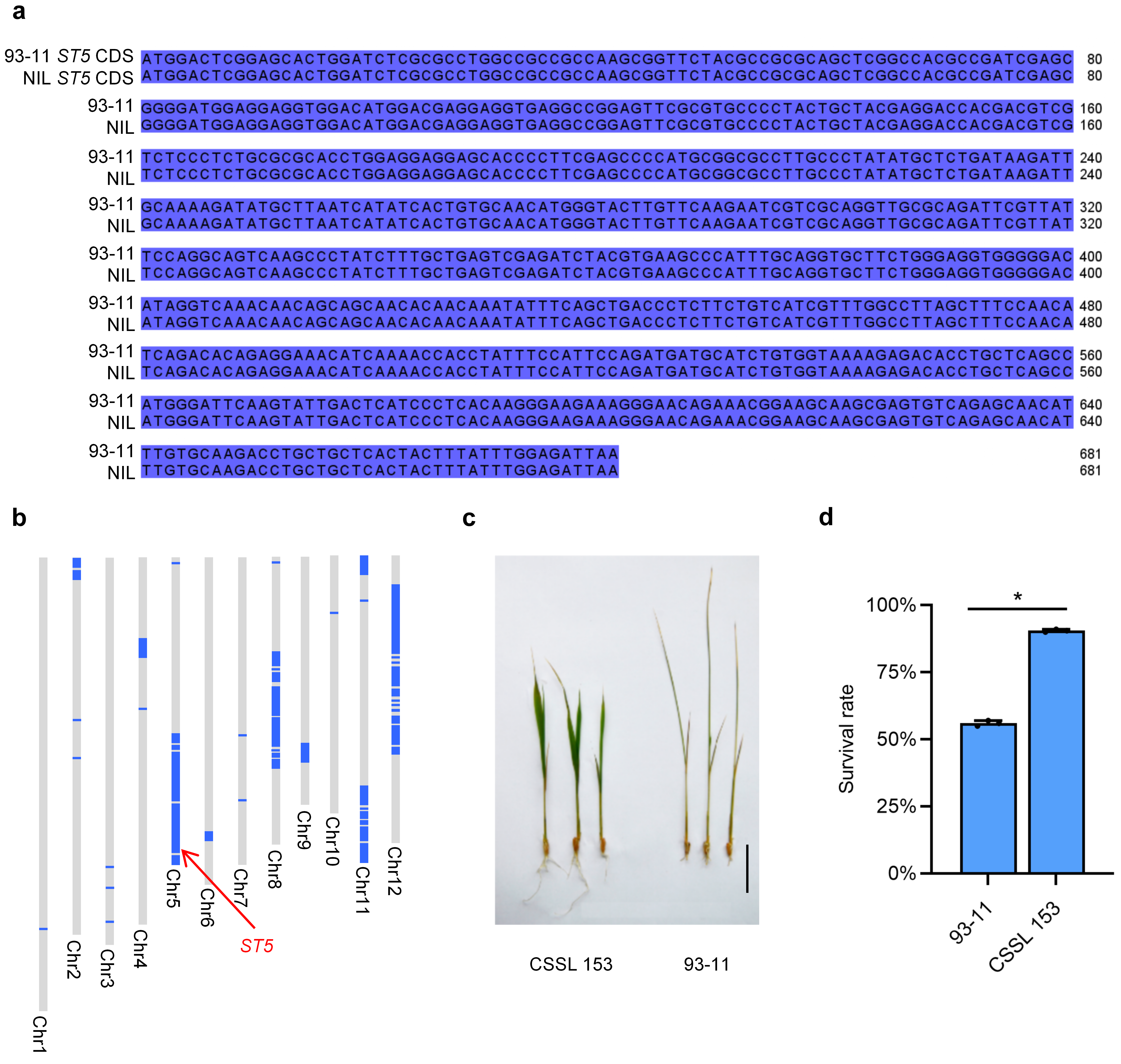
**Figure S3. CSSL153 is more salt tolerant than 93-11.**

a) Sequence alignment results of *ST5* coding region in 93-11 and NIL.

b) The genotype of CSSL153. Grey rectangles represent segments of 93-11, blue rectangles represent segments of wild rice.

c,d) Salt tolerance phenotype (c) and survival rates (d) of 93-11 and CSSL153. Two weeks seedlings were treated with 150 mM NaCl for seven days and recovered for seven days, then survival rate was calculated. Data are means ± SD (*n* = 3 biological replicates). *, *P* < 0.05. Scale bars = 2.5 cm.


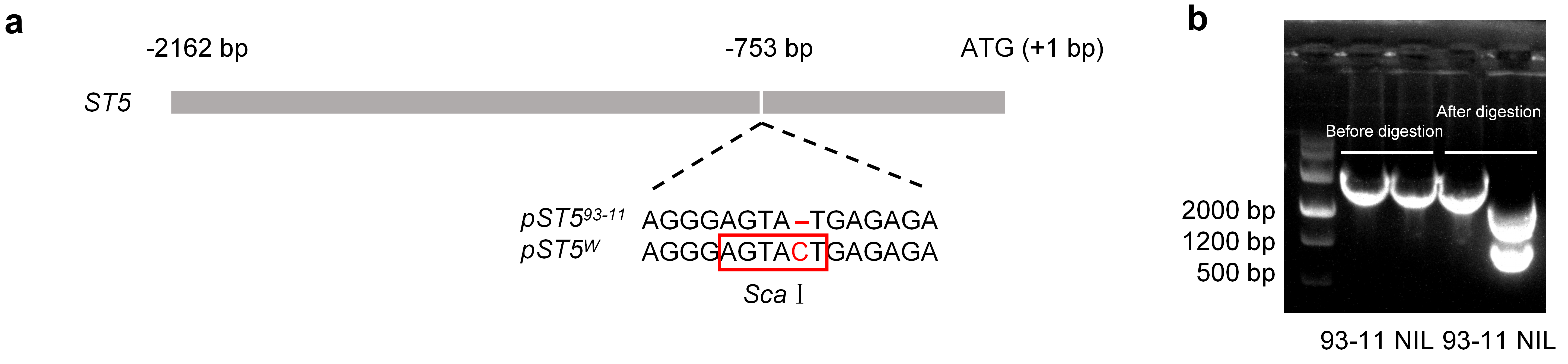
**Figure S4. Development of CAPS molecular markers for *ST5*.**

a) Alignments of two alleles of *ST5* promoter in 93-11 and the NIL. The SNP and enzyme digest sites for the CAPS marker were shown.

b) Electrophoresis shows CAPS marker polymorphism for *ST5* promoter. The CAPS marker was developed based on the -/C variation at site -753 bp of the promoter region between 93-11 and the NIL, and the restriction enzymes was *Sca* I. The full length of the promoter was 2,162-bp.


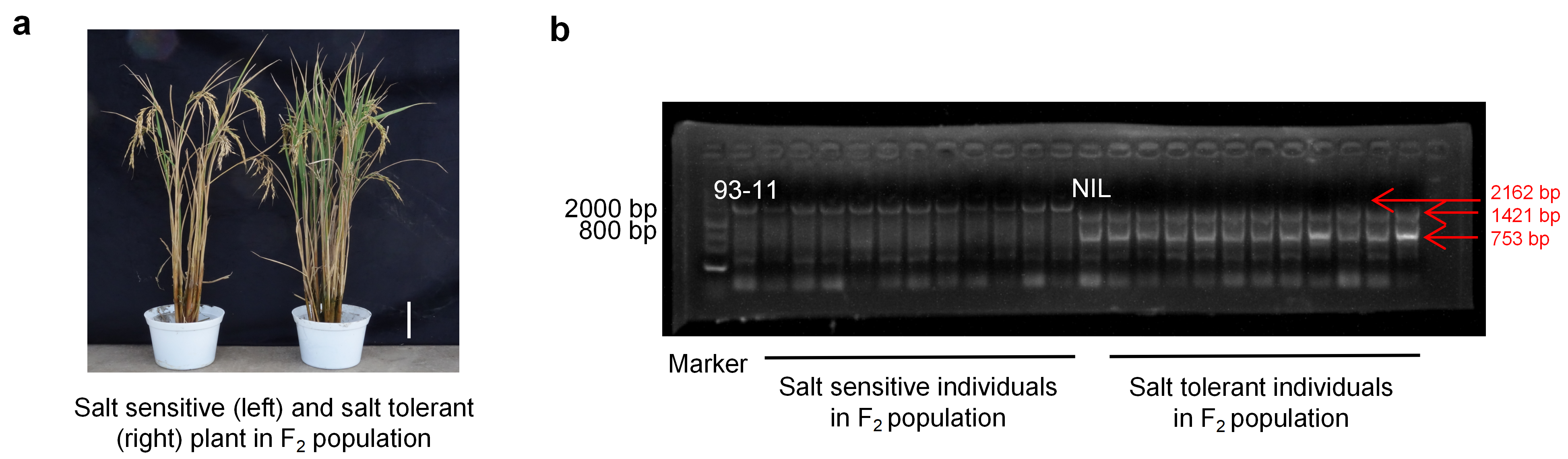


**Figure S5. Identification of phenotype and genotype of individual plants of 93-11/NIL F_2_ segregating population.**

a) Phenotype of two individuals from the 93-11/NIL F_2_ segregating population in 150 mM NaCl saline field. Scale bars = 10 cm.

b) Electrophoresis shows CAPS marker polymorphism for *ST5* promoter and identification of the *ST5* promoter types of salt tolerant and sensitive individuals in F_2_ population. The PCR product amplified from *pST5^W^* can be cleaved by *Sca* I enzyme, which produces DNA fragments of 1,421-bp and 753-bp. While that amplified from *pST5^93-11^* cannot be digested by *Sca* I enzyme, leading to a single band of 2,162-bp.


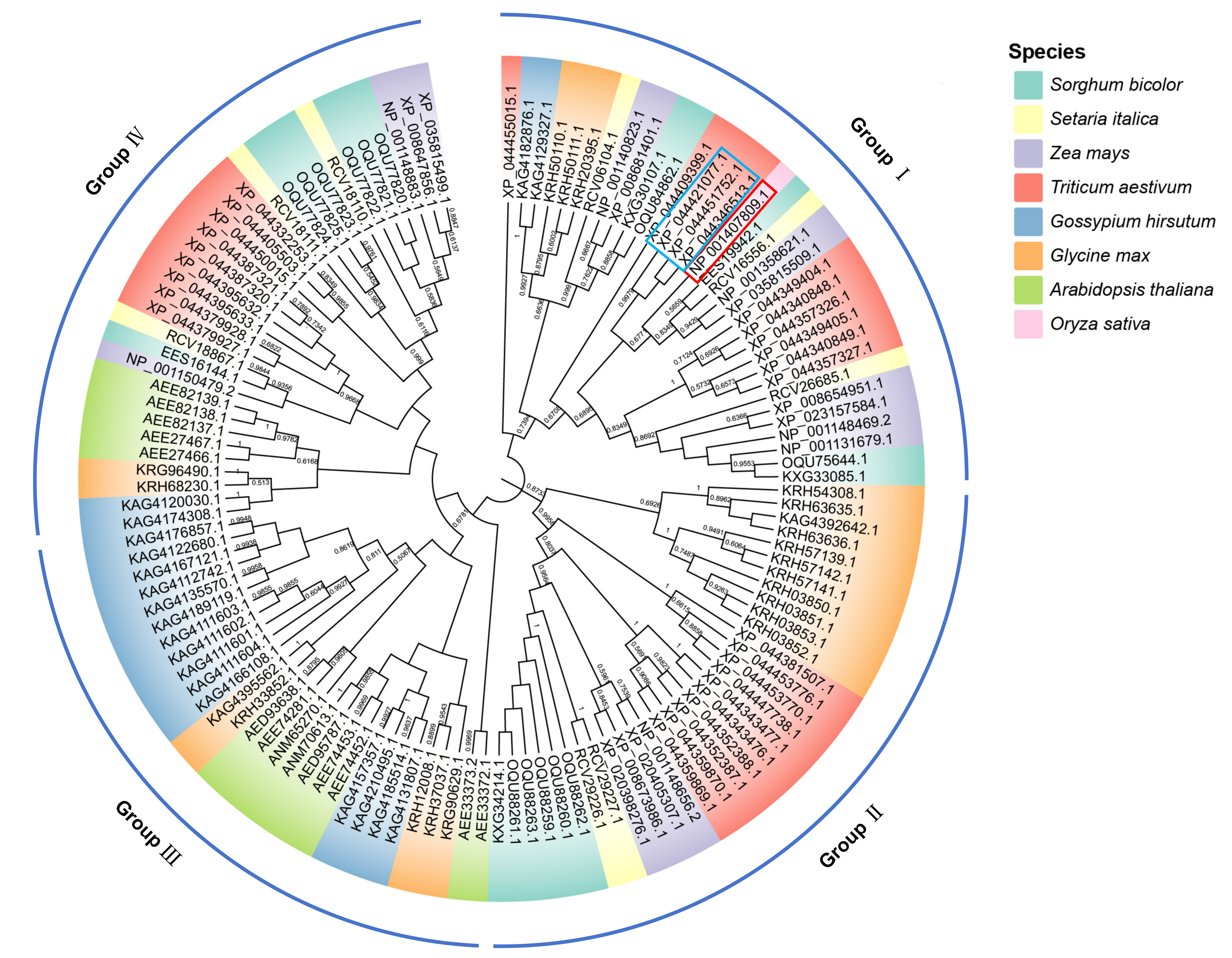
**Figure S6. Phylogenetic relationships of the ST5 family proteins.**

Phylogenetic tree was generated for ST5 homologous full protein sequences from *Arabidopsis thaliana*, *Sorghum bicolor*, *Setaria italica*, *Zea mays*, *Triticum aestivum*, *Gossypium hirsutum*, and *Glycine max* using MEGA12 (https://www.megasoftware.net/) with the neighbor-joining algorithms. Groups I-IV indicate the four groups of plant ST5 proteins. Bootstrap analysis was performed using 1000 replicates in MEGA12 to evaluate the reliability of the different phylogenetic groups. ST5 is highlighted with a red box. Three copys of TaDi19-1 are highlighted with a blue box.


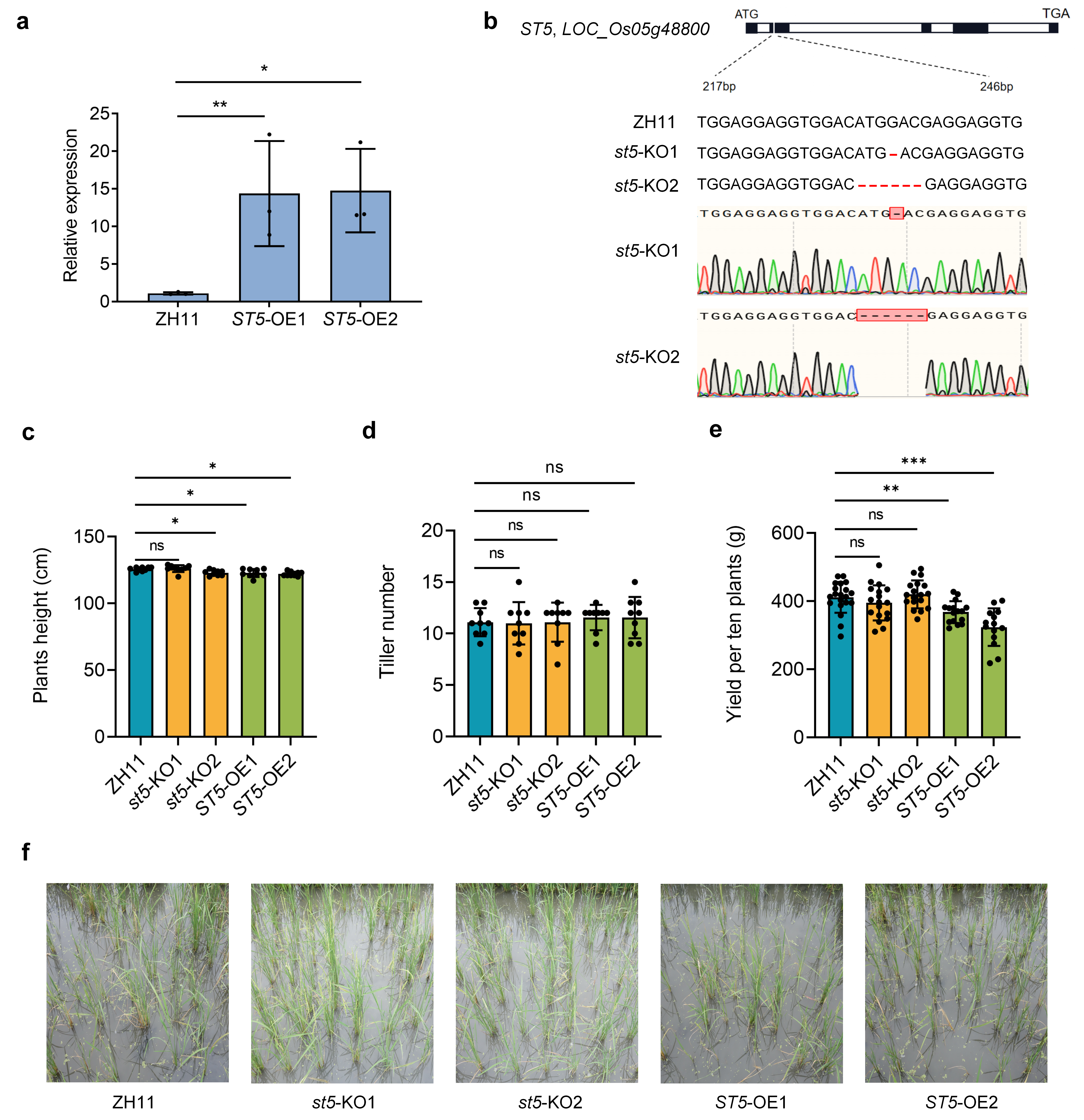


**Figure S7. Identification of *ST5* mutants and transgenic lines in ZH11.**

a) Expression levels of *ST5* in transgenic lines (*ST5*-OE1, *ST5*-OE2) of ZH11 background.

b) The sequencing results of mutations in *st5*-KO1 and *st5*-KO2. Black rectangles represent exons of *ST5*.

c-e) Investigation of agronomic traits of plant height (c), tiller number (d) and yield per ten plants (e) in the fields under normal growth conditions.

f) Phenotype of ZH11, *st5*-KO, and *ST5*-OE in 51 mM NaCl saline field.

Data are means ± SD (*n* ≥ 3). *, *P* < 0.05; **, *P* < 0.01; ***, *P* < 0.001; ns, no significant difference; Two-tailed Student’s *t*-test.


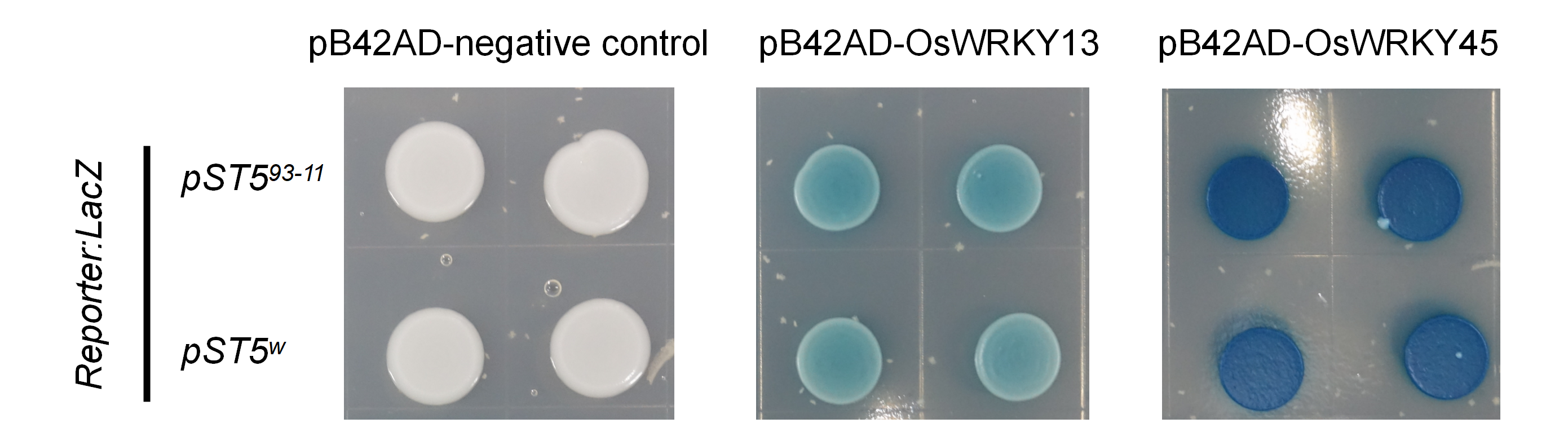


**Figure S8.** **OsWRKY13 and OsWRKY45 directly binds to the *pST5^93-11^* and *pST5^W^* in Y1H assays.**


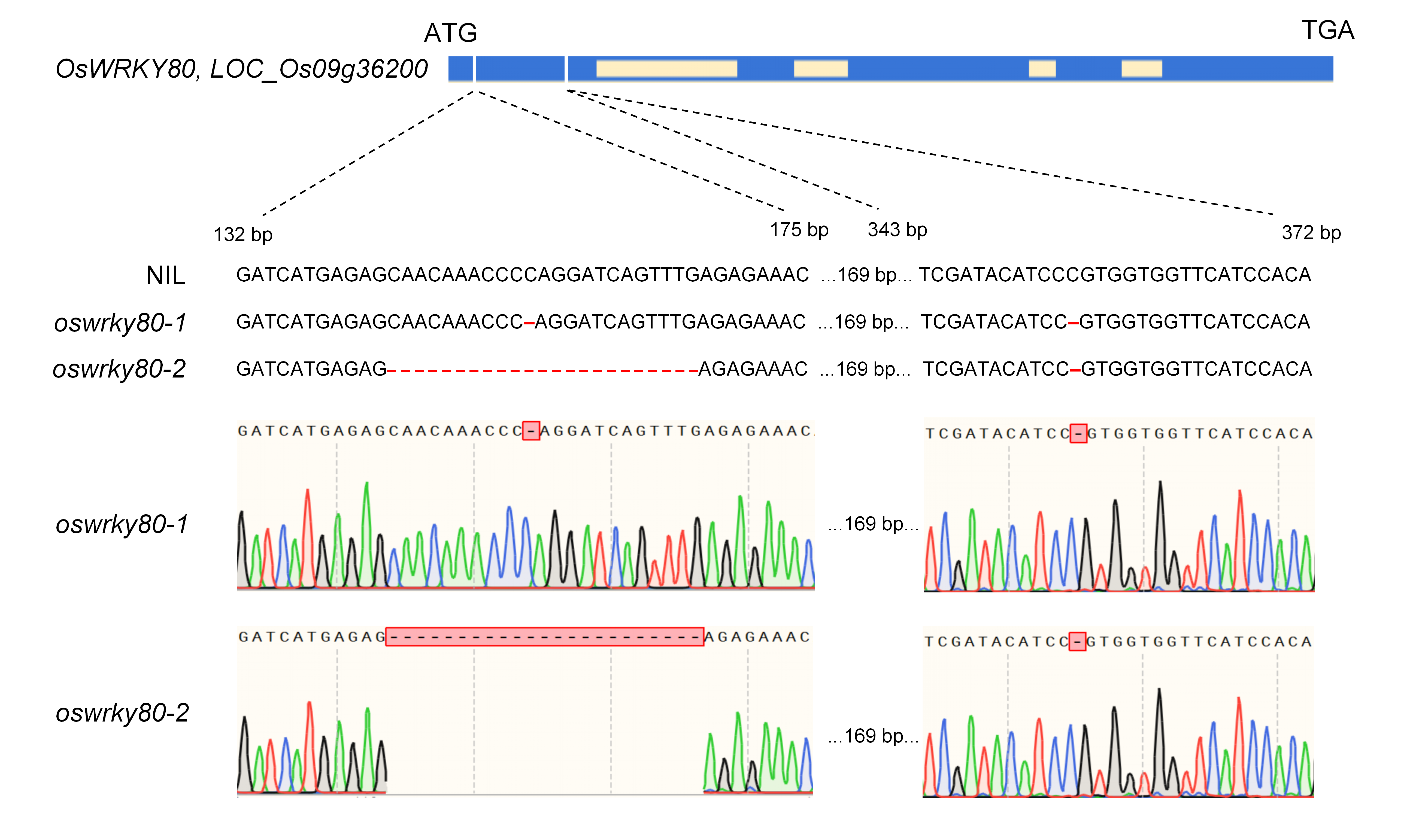
**Figure S9.** **Identification of *OsWRKY80* knock out lines in NIL background.** Alignment of *OsWRKY80* sequences for *oswrky80*-1, *oswrky80-2* and wild type NIL. Mutation sites were shown. Blue rectangles represent exons of *OsWRKY80*.


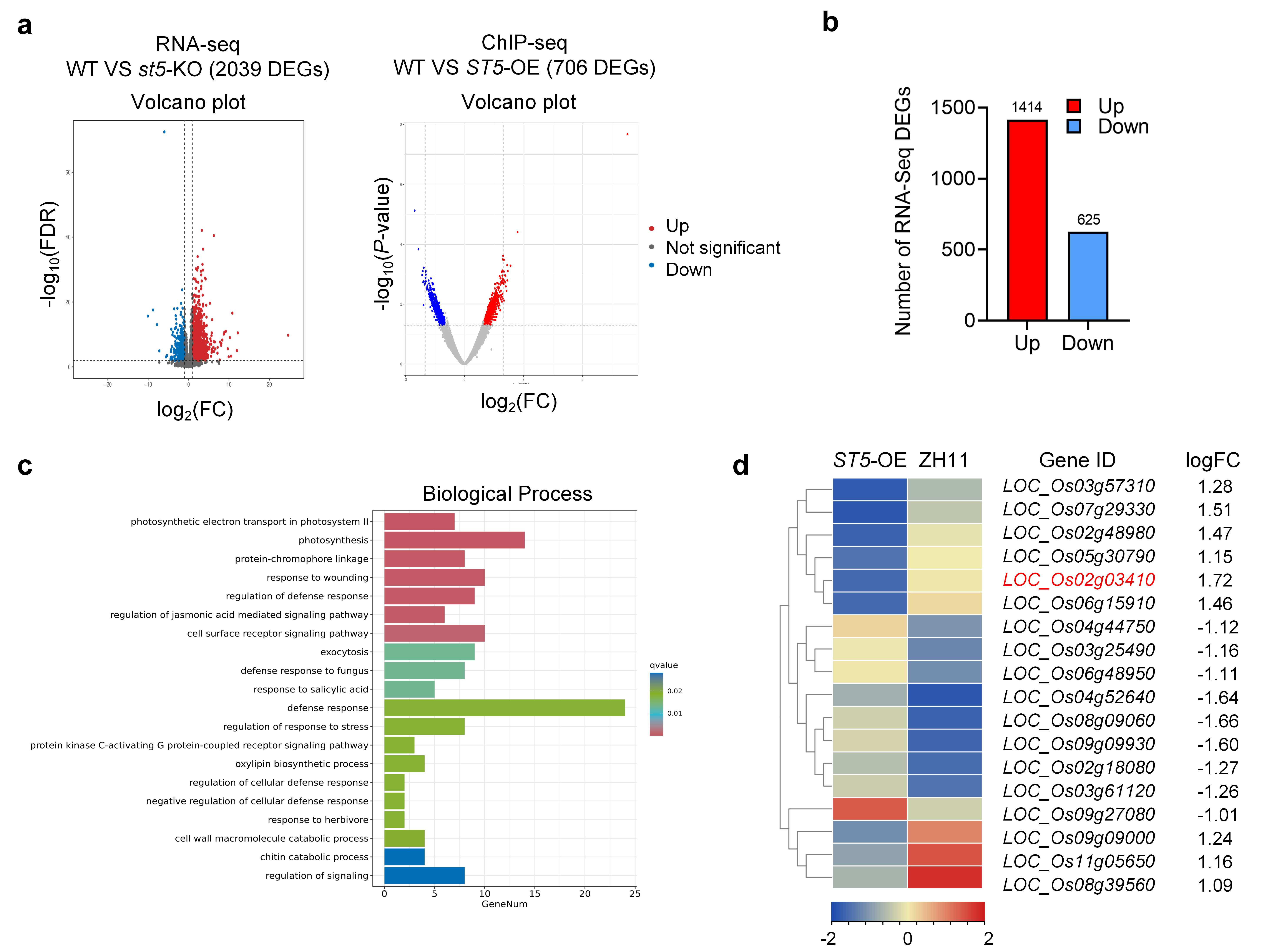
**Figure S10.** **RNA-seq and ChIP-seq showing genes regulated by ST5.**

a) Volcano plots showing fold changes of DEGs detected by RNA-seq between ZH11 and *st5*-KO, and ChIP-seq target genes between ZH11 and *ST5-*OE. Red and blue dots represent up- and down-regulated genes, respectively (*P*  <  0.01, absolute fold change > 2.0).

b) Distribution of up-regulated and down-regulated gene numbers detected by RNA-seq.

c) Gene ontology analysis of biological pathways enriched in the up-regulated genes identified through RNA-seq analysis. *q* value was computed by hypergeometric test.

d) Heatmap of 18 genes overlapped by RNA-seq and ChIP-seq. *OsCPK4* was highlighted. Values were standardized using Z-score.


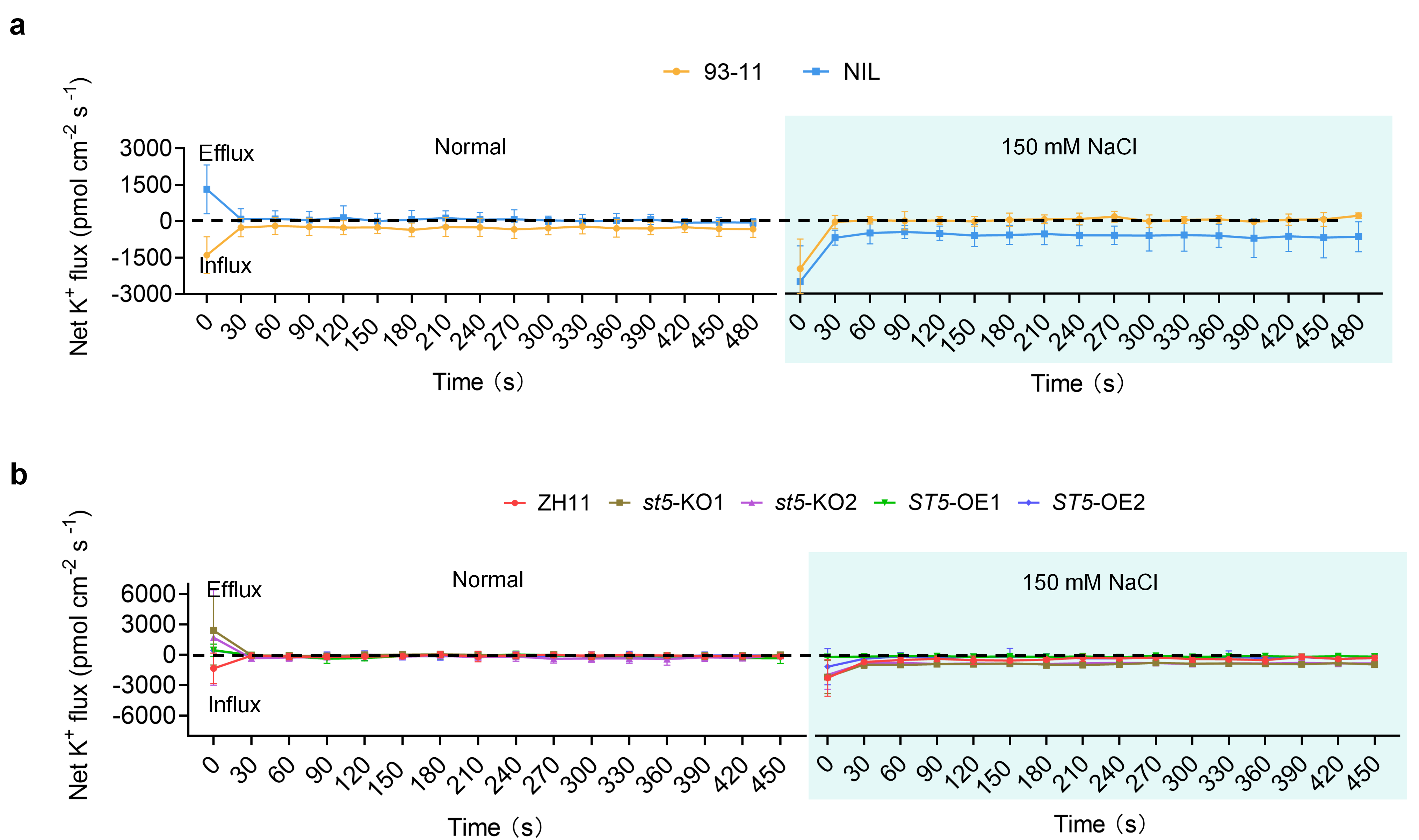


**Figure S11. Detection of K^+^ fluxes by NMT.**

a) Net K^+^ fluxes in 500 µm distance from root apex of 93-11, and the NIL

b) Net K^+^ fluxes in 500 µm distance from root apex of ZH11, *ST5*-OE, and *st5*-KO.

Measurements performed under 150 mM NaCl treatment. *n* = 3 biological replicates, data were presented as means ± SD.


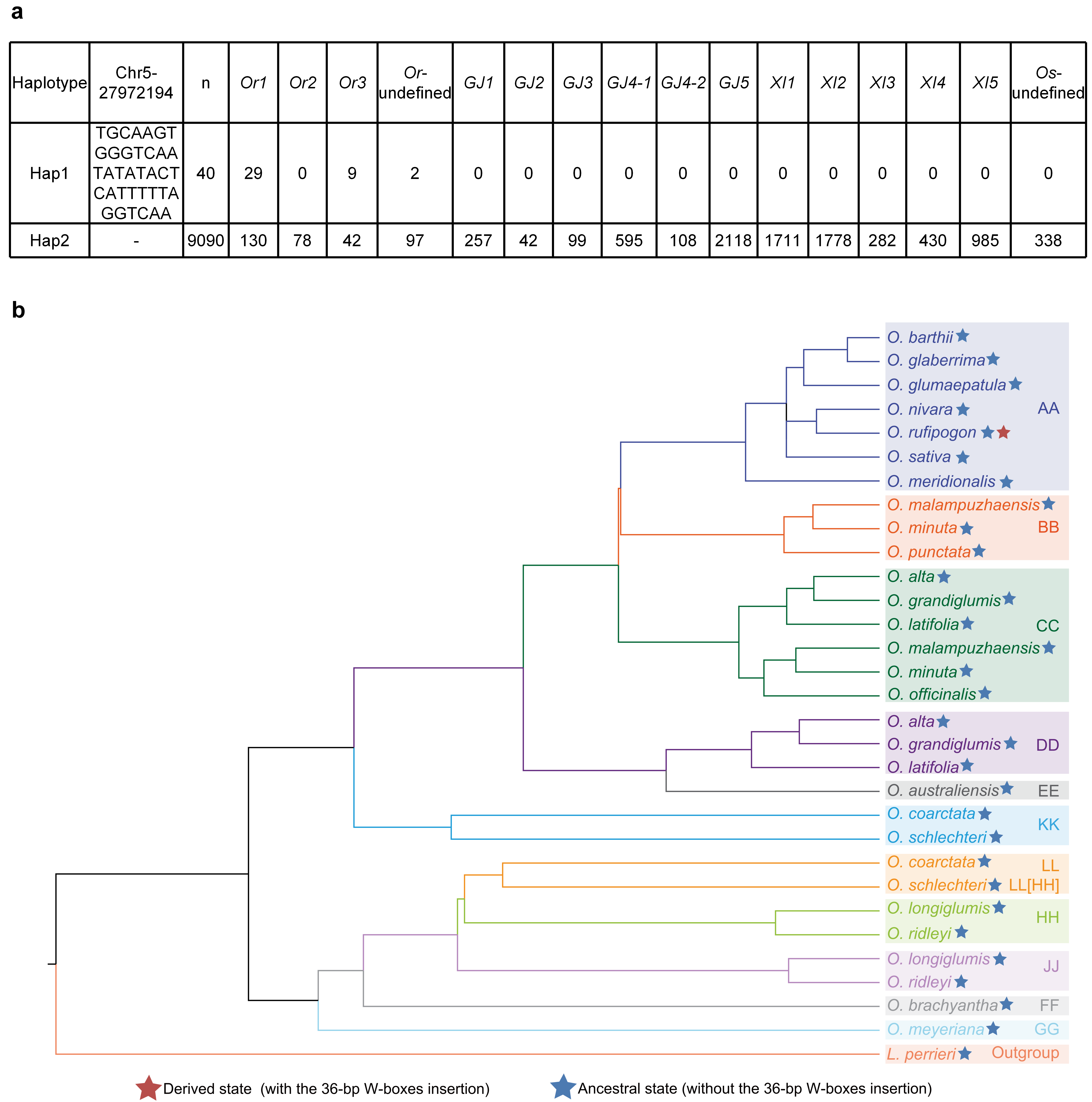


**Figure S12. Haplotype and evolutionary analysis of *ST5.***

a) Haplotype analysis of the 36-bp InDel of *ST5* based on a global gene pool of 10, 548 rice accessions. n, number of accessions. *Or*, *GJ* and *XI* refer to *O. rufipogon*, *O. sativa japonica* and *O. sativa indica* respectively. Samples with no sequencing data or heterozygous genotypes at this locus in the variant dataset were excluded.

b) The presence of the 36-bp InDel of *ST5* across the *Oryza* genomes.


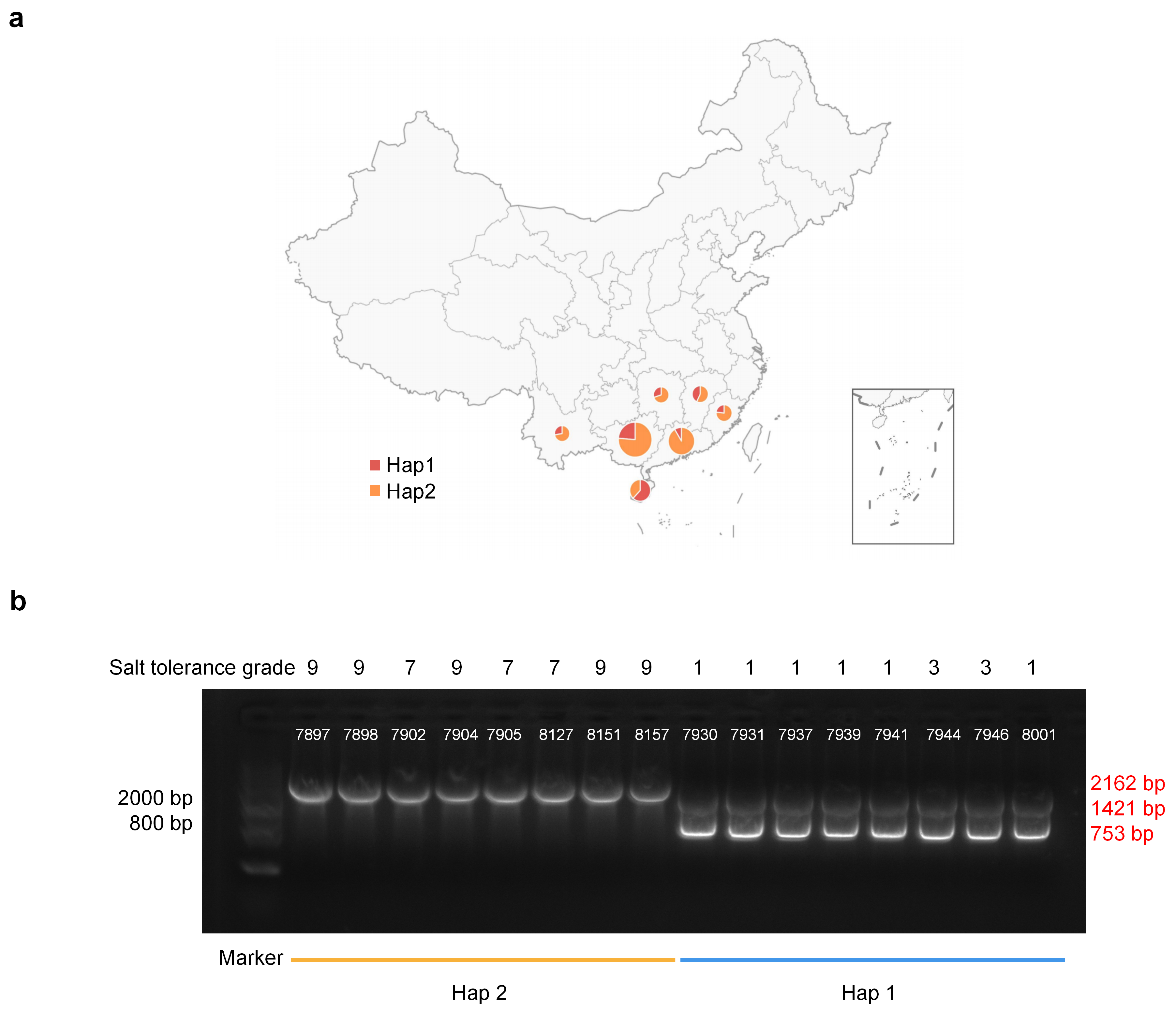


**Figure S13. Geographical distribution and genotype identification of 530 *O.* *rufipogon* accessions in China.**

a) The geographical distribution of Hap1 and Hap2 in *O. rufipogon*. The size of the pie represents the number of accessions within the location.

b) Electrophoresis shows CAPS marker polymorphism for *ST5* in *O. rufipogon*. Hap1 type can be cleaved by *Sca* I enzyme, which produced DNA fragments of 1,421-bp and 753-bp. Hap2 type cannot be digested by *Sca* I enzyme, leading to a single band of 2,162-bp. The number above each DNA band represents the accession number of wild rice materials, corresponding to the numbers in Fig. 5f. The salt tolerance grade of each accession were marked above the gel image.


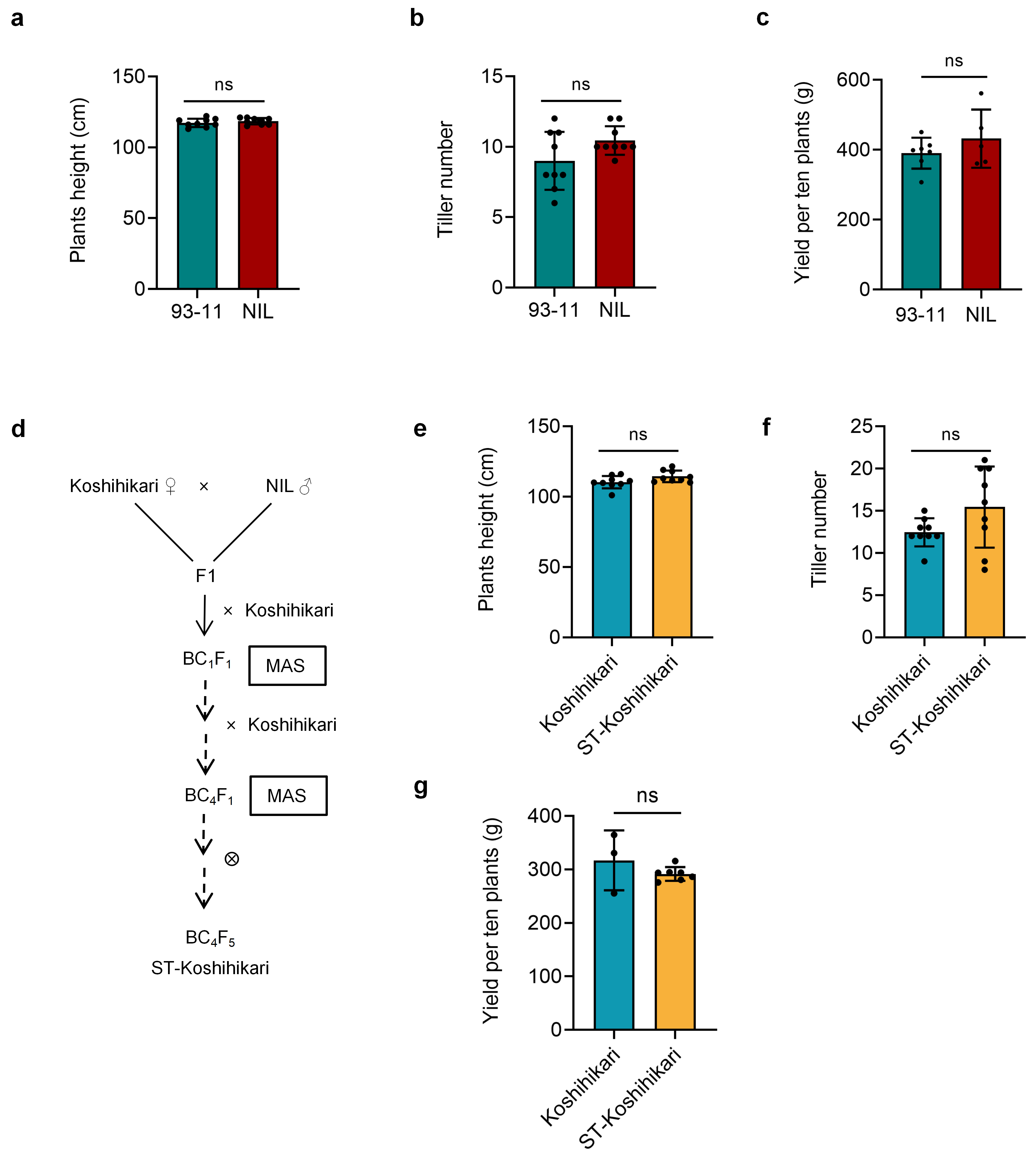


**Figure S14. Field agronomic traits of 93-11, NIL, Koshihikari, and ST-Koshihikari under normal conditions.**

a-c) Field agronomic trials for 93-11 and NIL of plant height (a), tiller number (b) and yield per ten plants (c) under normal growth conditions.

d) Schematic of the development of ST-Koshihikari with marker assisted selection (MAS).

e-g) Field agronomic trials for Koshihikari and ST-Koshihikari of plant height (e), tiller number (f) and yield per ten plants (g) under normal growth conditions.

Data are means ± SD (*n* ≥ 3). *, *P* < 0.05; **, *P* < 0.01; ***, *P* < 0.001; ns, no significant difference; Two-tailed Student’s *t*-test.
