# Supplementary material for: A Rare Allele of ST5 From Wild Rice Enhances Salt Tolerance in Rice
Source: Adv Sci (Weinh). 2026 May 28;13(43):e16159. doi: 10.1002/advs.202516159 (PMC13336088; doi:10.1002/advs.202516159)
Supplement: Supplementary file 2 — Supporting File 2: advs75600‐sup‐0002‐TableS1‐S6.docx. [file ADVS-13-e16159-s001.docx]

**Table S1. The CSSL salt treatment phenotype data**

| Indices | Parent | CSSLs |  |  |
| --- | --- | --- | --- | --- |
|  | 93-11 | Min | Max | Mean |
| Germination rate (%) | 4.7222±2.48 | 0.0000 | 74.2319 | 24.1200 |
| Seedling survival rate (%) | 54.1667±6.80 | 16.6667 | 88.8889 | 55.4071 |
| Seedling Salt-tolerant Grade | 5 | 9 | 1 | 6.6751 |
| Seedling Survival Days (d) | 8.7±0.02 | 6.6 | 11.5 | 9.3 |
| Seedling Dry weight of the grade (g) | 0.8467±0.10 | 0.5644 | 1.5177 | 0.9992 |
| Seedling Dry weight of underground (g) | 0.8304±0.03 | 0.5530 | 1.3656 | 1.0000 |

**Table S2. Relevance of salt tolerance index in seedling stage**

|  | Seedling survival rate | Seedling Salt-tolerant Grade | Seedling Survival Days | Seedling Dry weight of the grade | Seedling Dry weight of underground |
| --- | --- | --- | --- | --- | --- |
| Seedling survival rate | 1 | 0.086 | 0.042 | 0.045 | -0.032 |
| Seedling Salt-tolerant Grade |  | 1 | 0.639^**^ | -0.071 | 0.03 |
| Seedling Survival Days |  |  | 1 | 0.159^*^ | 0.167^*^ |
| Seedling Dry weight of the grade |  |  |  | 1 | 0.728^**^ |
| Seedling Dry weight of underground |  |  |  |  | 1 |

**Table S3. Salt-tolerant QTLs of CSSL (SSR/InDel and SNP marker)**

| **Treatments** | | **Chr.** | **Peak marker** | **Flanking markers** | **LOD** | **PVE (%)** | **Add effect** | **QTL name** |
| --- | --- | --- | --- | --- | --- | --- | --- | --- |
| Germination rate | | | | | | | |  |
|  |  | 4 | S4_22283843 | S4_22198050 - S4_22358656 | 2.6176 | 4.5976 | 0.0668 | *qGR4.1* |
|  |  | 7 | S7_23160626 | S7_23077850 - S7_23293939 | 6.0353 | 11.0283 | 0.1168 | *qGR7.1* |
|  |  | 11 | InDel11-4 | InDel11-3 - InDel11-5 | 2.5524 | 5.9383 | 0.0731 | *qGR11.1* |
|  |  | 11 | S11_16987307 | S11_16849537 - S11_17292444 | 6.7571 | 12.4518 | 0.0760 | *qGR11.2* |
| Seedling survival rate | | | | | | | |  |
|  |  | 2 | InDel2-10 | RM475 - RM6318 | 3.9369 | 8.7851 | 7.0447 | *qSSR2.1* |
|  |  | 2 | S2_1049672 | S2_989128 - S2_1227422 | 4.2003 | 6.3572 | 7.5950 | *qSSR2.2* |
|  |  | 2 | S2_24278397 | S2_24128850 - S2_24387301 | 3.6938 | 5.5573 | 6.3315 | *qSSR2.3* |
|  |  | 5 | InDel5-10 | RM598 – RM534 | 3.9455 | 8.8056 | 7.9058 | *qSSR5.1* |
|  |  | 5 | S5_27886769 | S5_27733816 - S5_27973430 | 5.6221 | 8.6531 | 6.8374 | *qSSR5.1* |
|  |  | 6 | S6_29003916 | S6_28999115 - S6_29194924 | 7.3310 | 11.5132 | 11.9654 | *qSSR6.1* |
| Seedling Salt-tolerant Grade | | | | | | | |  |
|  |  | 2 | InDel2-5 | RM452 - RM324 | 2.7520 | 6.4857 | 1.0106 | *qSSG2.1* |
|  |  | 5 | S5_27886769 | S5_27733816 - S5_27973430 | 3.6025 | 8.1340 | 0.7271 | *qSSG5.1* |
|  |  | 8 | S8_9390178 | S8_9291925 - S8_9484528 | 2.8483 | 6.3748 | 0.8962 | *qSSG8.1* |
| Seedling Survival Days | | | | | | | |  |
|  |  | 1 | InDel1-16 | RM128 - RM472 | 3.0878 | 7.2669 | -0.4618 | *qSSD1.1* |
|  |  | 1 | S1_38368329 | S1_38292070 - S1_38409107 | 6.0902 | 9.7907 | -0.4110 | *qSSD1.2* |
|  |  | 4 | S4_35501387 | S4_35469308 - | 2.6568 | 4.0973 | -0.2387 | *qSSD4.1* |

**Table S3. (Continued)**

| **Treatments** | | **Chr.** | **Peak marker** | **Flanking markers** | **LOD** | **PVE (%)** | **Add effect** | **QTL name** |
| --- | --- | --- | --- | --- | --- | --- | --- | --- |
|  |  | 6 | S6_4286559 | S6_3658340 - S6_4375518 | 2.9775 | 4.6085 | 0.3703 | *qSSD6.1* |
|  |  | 8 | RM339 | InDel8-7 - InDel8-8 | 2.9269 | 6.8725 | 0.3344 | *qSSD8.1* |
|  |  | 8 | S8_2689486 | S8_2513855 - S8_2786791 | 6.6480 | 10.7585 | 0.4111 | *qSSD8.2* |
|  |  | 11 | S11_24264059 | S11_24103036 - S11_24349408 | 3.4826 | 5.4272 | -0.5131 | *qSSD11.1* |
| Seedling Dry weight of underground | | | | | | | |  |
|  |  | 1 | S1_35391843 | S1_35267727 - S1_35428764 | 5.1812 | 7.3688 | 0.0764 | *qUDW1.1* |
|  |  | 1 | S1_38409107 | S1_38368329 - S1_38524922 | 3.1496 | 4.3755 | -0.0854 | *qUDW1.2* |
|  |  | 11 | InDel11-5 | InDel11-4 - RM206 | 2.9929 | 6.9270 | 0.0729 | *qUDW11.1* |
|  |  | 11 | S11_14555474 | S11_14492837 - S11_14682925 | 4.4734 | 6.3103 | 0.0779 | *qUDW11.2* |

**Table S4. Sequence variations in the *qST5* locus between 93-11 and wild rice**

| **Gene Name** | **Position** | **93-11** | **Wild rice** |
| --- | --- | --- | --- |
| *LOC_Os05g48740* | Chr5_28698022 | AAA | --- |
|  | Chr5_28698043 | GAAGAA | ------ |
|  | Chr5_28698641 | -- | CA |
|  | Chr5_28698838 | -- | AA |
|  | Chr5_28699096 | ---------- | TGTTGTTGCT |
|  | Chr5_28699286 | G | A |
|  | Chr5_28699336 | C | A |
|  | Chr5_28699340 | T | C |
|  | Chr5_28699364 | -- | GA |
|  | Chr5_28699410 | C | T |
|  | Chr5_28699551 | GA | TG |
| *LOC_Os05g48750* | Chr5_28701350 | G | T |
|  | Chr5_28703602 | C | T |
|  | Chr5_28703643 | T | C |
|  | Chr5_28703774 | A | - |

**Table S4. (Continued)**

| **Gene Name** | **Position** | **93-11** | **Wild rice** |
| --- | --- | --- | --- |
| *LOC_Os05g48750* | Chr5_28703883 | T | C |
|  | Chr5_28704014 | A | - |
|  | Chr5_28704635 | G | A |
|  | Chr5_28704721 | G | A |
|  | Chr5_28705029 | C | T |
|  | Chr5_28705095 | C | G |
|  | Chr5_28705174 | A | G |
|  | Chr5_28705189 | C | A |
|  | Chr5_28705204 | T | A |
|  | Chr5_28705263 | T | C |
|  | Chr5_28705387 | G | A |
|  | Chr5_28705428 | G | A |
|  | Chr5_28705447 | T | - |
|  | Chr5_28705455 | A | T |
|  | Chr5_28705499 | G | A |

**Table S4. (Continued)**

| **Gene Name** | **Position** | **93-11** | **Wild rice** |
| --- | --- | --- | --- |
| *LOC_Os05g48750* | Chr5_28705553 | C | T |
|  | Chr5_28705678 | - | T |
| *LOC_Os05g48760* | Chr5_28706497 | T | C |
|  | Chr5_28706538 | G | A |
|  | Chr5_28706556 | G | A |
|  | Chr5_28706578 | A | G |
|  | Chr5_28706741 | T | C |
|  | Chr5_28706776 | A | G |
|  | Chr5_28706802 | T | C |
|  | Chr5_28706812 | A | G |
|  | Chr5_28706892 | T | C |
|  | Chr5_28706895 | A | G |
|  | Chr5_28706905 | G | A |
|  | Chr5_28706911 | A | G |
|  | Chr5_28707012 | A | T |

**Table S4. (Continued)**

| **Gene Name** | **Position** | **93-11** | **Wild rice** |
| --- | --- | --- | --- |
| *LOC_Os05g48760* | Chr5_28707058 | G | A |
|  | Chr5_28707122 | CG | TA |
|  | Chr5_28707160 | GG | TA |
|  | Chr5_28707172 | T | C |
|  | Chr5_28707256 | T | A |
|  | Chr5_28707294 | C | T |
|  | Chr5_28707358 | T | C |
|  | Chr5_28707377 | T | C |
|  | Chr5_28707380 | G | A |
|  | Chr5_28707434 | A | G |
|  | Chr5_28707557 | C | T |
|  | Chr5_28707572 | C | A |
|  | Chr5_28707598 | A | G |
|  | Chr5_28707607 | A | G |
|  | Chr5_28707717 | A | T |

**Table S4. (Continued)**

| **Gene Name** | **Position** | **93-11** | **Wild rice** |
| --- | --- | --- | --- |
| *LOC_Os05g48760* | Chr5_28707761 | A | G |
|  | Chr5_28707783 | T | C |
|  | Chr5_28707901 | T | C |
|  | Chr5_28707968 | A | T |
|  | Chr5_28708140 | - | A |
|  | Chr5_28708190 | G | A |
|  | Chr5_28708550 | --- | CTC |
|  | Chr5_28710992 | A | C |
|  | Chr5_28711097 | A | C |
|  | Chr5_28711121 | G | A |
|  | Chr5_28711178 | A | C |
|  | Chr5_28711207 | T | C |
|  | Chr5_28711236 | C | T |
|  | Chr5_28711497 | G | A |
|  | Chr5_28711510 | A | T |

**Table S4. (Continued)**

| **Gene Name** | **Position** | **93-11** | **Wild rice** |
| --- | --- | --- | --- |
| *LOC_Os05g48760* | Chr5_28711657 | C | A |
| *LOC_Os05g48770* | Chr5_28715482 | T | C |
|  | Chr5_28715677 | T | G |
|  | Chr5_28716667 | T | A |
|  | Chr5_28717837 | T | G |
|  | Chr5_28717936 | C | T |
|  | Chr5_28718058 | G | A |
|  | Chr5_28718061 | ---- | AAAA |
|  | Chr5_28718095 | G | T |
|  | Chr5_28718149 | - | T |
|  | Chr5_28718195 | ACGTATATAGGCTCCTCGAAA | --------------------- |
|  | Chr5_28718272 | A | - |
|  | Chr5_28718353 | C | T |
|  | Chr5_28718390 | T | C |
|  | Chr5_28718413 | C | T |

**Table S4. (Continued)**

| **Gene Name** | **Position** | **93-11** | **Wild rice** |
| --- | --- | --- | --- |
| *LOC_Os05g48770* | Chr5_28718423 | T | C |
|  | Chr5_28718433 | T | C |
|  | Chr5_28718425 | T | C |
|  | Chr5_28718441 | GT | CC |
|  | Chr5_28718470 | T | A |
|  | Chr5_28718494 | T | A |
|  | Chr5_28718498 | A | G |
|  | Chr5_28718516 | T | A |
|  | Chr5_28718524 | A | - |
|  | Chr5_28718564 | A | G |
|  | Chr5_28718571 | ---- | GCTA |
|  | Chr5_28718581 | G | T |
|  | Chr5_28718666 | G | T |
|  | Chr5_28718682 | G | A |
|  | Chr5_28718689 | A | G |

**Table S4. (Continued)**

| **Gene Name** | **Position** | **93-11** | **Wild rice** |
| --- | --- | --- | --- |
| *LOC_Os05g48770* | Chr5_28718695 | C | T |
|  | Chr5_28718698 | AT | GG |
|  | Chr5_28718719 | G | A |
|  | Chr5_28718722 | T | G |
|  | Chr5_28718726 | A | G |
|  | Chr5_28718854 | A | G |
|  | Chr5_28718964 | A | C |
|  | Chr5_28719014 | G | A |
|  | Chr5_28719077 | C | T |
|  | Chr5_28719089 | G | A |
|  | Chr5_28719101 | A | G |
|  | Chr5_28719179 | G | A |
|  | Chr5_28719181 | T | C |
|  | Chr5_28719183 | C | T |
|  | Chr5_28719187 | A | G |

**Table S4. (Continued)**

| **Gene Name** | **Position** | **93-11** | **Wild rice** |
| --- | --- | --- | --- |
| *LOC_Os05g48770* | Chr5_28719218 | G | T |
|  | Chr5_28719222 | G | A |
|  | Chr5_28719260 | A | G |
|  | Chr5_28719292 | G | A |
|  | Chr5_28719305 | A | G |
|  | Chr5_28719327 | C | T |
|  | Chr5_28719712 | A | G |
|  | Chr5_28719716 | G | A |
|  | Chr5_28719719 | T | C |
|  | Chr5_28719804 | C | T |
|  | Chr5_28719837 | A | G |
|  | Chr5_28719840 | C | G |
|  | Chr5_28719848 | G | A |
|  | Chr5_28719850 | G | T |
|  | Chr5_28719876 | C | T |

**Table S4. (Continued)**

| **Gene Name** | **Position** | **93-11** | **Wild rice** |
| --- | --- | --- | --- |
| *LOC_Os05g48770* | Chr5_28719908 | A | G |
|  | Chr5_28719942 | T | C |
|  | Chr5_28719957 | T | C |
|  | Chr5_28719960 | C | T |
|  | Chr5_28719962 | G | A |
|  | Chr5_28719969 | C | A |
|  | Chr5_28719980 | ------ | CTCGTG |
|  | Chr5_28719984 | G- | AC |
|  | Chr5_28719987 | ---- | TAAA |
|  | Chr5_28719993 | A | G |
|  | Chr5_28720005 | --- | TTA |
| *LOC_Os05g48780* | Chr5_28720061 | A | G |
|  | Chr5_28720072 | A | G |
|  | Chr5_28720080 | G | A |
|  | Chr5_28720131 | - | A |

**Table S4. (Continued)**

| **Gene Name** | **Position** | **93-11** | **Wild rice** |
| --- | --- | --- | --- |
| *LOC_Os05g48780* | Chr5_28720167 | A | C |
|  | Chr5_28720169 | C | T |
|  | Chr5_28720191 | C | A |
|  | Chr5_28720203 | G | A |
|  | Chr5_28720211 | G | A |
|  | Chr5_28720234 | C | T |
|  | Chr5_28720251 | A | G |
|  | Chr5_28720269 | A | G |
|  | Chr5_28720299 | A | C |
|  | Chr5_28720315 | G | C |
|  | Chr5_28720327 | T | C |
|  | Chr5_28720365 | G | A |
|  | Chr5_28720367 | C | T |
|  | Chr5_28720380 | A | G |
|  | Chr5_28720390 | G | A |

**Table S4. (Continued)**

| **Gene Name** | **Position** | **93-11** | **Wild rice** |
| --- | --- | --- | --- |
| *LOC_Os05g48780* | Chr5_28720428 | G | A |
|  | Chr5_28720434 | C | T |
|  | Chr5_28720440 | G | A |
|  | Chr5_28720449 | T | A |
|  | Chr5_28720492 | A | G |
|  | Chr5_28720520 | C | T |
|  | Chr5_28720522 | T | C |
|  | Chr5_28720564 | G | A |
|  | Chr5_28720585 | A | C |
|  | Chr5_28720589 | G | - |
|  | Chr5_28720614 | T | C |
|  | Chr5_28720667 | C | A |
|  | Chr5_28720810 | G | A |
|  | Chr5_28720822 | T | C |
|  | Chr5_28720837 | G | A |

**Table S4. (Continued)**

| **Gene Name** | **Position** | **93-11** | **Wild rice** |
| --- | --- | --- | --- |
| *LOC_Os05g48780* | Chr5_28720856 | G | A |
|  | Chr5_28720910 | C | T |
|  | Chr5_28720917 | T | C |
|  | Chr5_28720946 | A | G |
|  | Chr5_28720971 | C | A |
|  | Chr5_28720974 | C | T |
|  | Chr5_28721017 | A | G |
|  | Chr5_28721027 | A | G |
|  | Chr5_28721049 | G | C |
| *LOC_Os05g48790* | Chr5_28724574 | C | T |
|  | Chr5_28724580 | C | T |
|  | Chr5_28724588 | C | T |
|  | Chr5_28724623 | GTGTATATATATATGTGTGTGT | ---------------------- |
|  | Chr5_28724759 | G | A |
|  | Chr5_28724814 | G | A |

**Table S4. (Continued)**

| **Gene Name** | **Position** | **93-11** | **Wild rice** |
| --- | --- | --- | --- |
| *LOC_Os05g48790* | Chr5_28725125 | G | C |
|  | Chr5_28725440 | T | C |
|  | Chr5_28725484 | C | G |
|  | Chr5_28725536 | T | C |
|  | Chr5_28725569 | TTCTGTATG | --------- |
|  | Chr5_28726070 | C | T |
|  | Chr5_28727712 | G | A |
|  | Chr5_28727868 | A | G |
|  | Chr5_28727875 | G | A |
|  | Chr5_28727877 | TC | CT |
|  | Chr5_28727882 | A | G |
|  | Chr5_28727904 | - | G |
|  | Chr5_28727913 | G | C |
|  | Chr5_28727923 | A | G |
|  | Chr5_28727945 | A | G |

**Table S4. (Continued)**

| **Gene Name** | **Position** | **93-11** | **Wild rice** |
| --- | --- | --- | --- |
| *LOC_Os05g48790* | Chr5_28727959 | G | T |
|  | Chr5_28727966 | ATG | --- |
|  | Chr5_28727972 | - | T |
|  | Chr5_28727980 | - | A |
|  | Chr5_28727986 | G | A |
|  | Chr5_28727989 | A | G |
|  | Chr5_28728001 | T | G |
|  | Chr5_28728024 | T | A |
|  | Chr5_28728078 | A | - |
|  | Chr5_28728131 | AA | GG |
|  | Chr5_28728137 | A | G |
|  | Chr5_28728173 | T | A |
|  | Chr5_28728206 | GC | AT |
|  | Chr5_28728237 | T | C |
|  | Chr5_28728245 | A | T |

**Table S4. (Continued)**

| **Gene Name** | **Position** | **93-11** | **Wild rice** |
| --- | --- | --- | --- |
| *LOC_Os05g48790* | Chr5_28728267 | A | G |
|  | Chr5_28728283 | C | T |
|  | Chr5_28728311 | A | G |
|  | Chr5_28728359 | C | T |
|  | Chr5_28728421 | A | G |
|  | Chr5_28728446 | T | C |
|  | Chr5_28728458 | - | C |
|  | Chr5_28728465 | T | A |
|  | Chr5_28728579 | G | A |
|  | Chr5_28728640 | TGC | -CT |
|  | Chr5_28728701 | TGATTGACATTTTGCGT- | C |
|  | Chr5_28728758 | C | A |
|  | Chr5_28728834 | G | A |
|  | Chr5_28728881 | G | A |
|  | Chr5_28728987 | G | A |

**Table S4. (Continued)**

| **Gene Name** | **Position** | **93-11** | **Wild rice** |
| --- | --- | --- | --- |
| *LOC_Os05g48790* | Chr5_28729016 | A | G |
|  | Chr5_28729097 | T | C |
|  | Chr5_28729109 | T | C |
|  | Chr5_28729112 | T | C |
|  | Chr5_28729145 | G | A |
|  | Chr5_28729147 | C | T |
|  | Chr5_28729163 | A | G |
|  | Chr5_28729179 | G | A |
|  | Chr5_28729228 | C | T |
|  | Chr5_28729269 | T | C |
|  | Chr5_28729295 | T | C |
|  | Chr5_28729298 | G | C |
|  | Chr5_28729336 | T | C |
|  | Chr5_28729401 | T | C |
|  | Chr5_28729458 | T | C |

**Table S4. (Continued)**

| **Gene Name** | **Position** | **93-11** | **Wild rice** |
| --- | --- | --- | --- |
| *LOC_Os05g48810* | Chr5_28736465 | A | G |
|  | Chr5_28737487 | T | G |
|  | Chr5_28737600 | T | C |
|  | Chr5_28738423 | T | C |
|  | Chr5_28738423 | C | G |
|  | Chr5_28738498 | C | T |
|  | Chr5_28738524 | G | C |
|  | Chr5_28738537 | T | C |
|  | Chr5_28738613 | C | T |
|  | Chr5_28738616 | A | C |
|  | Chr5_28738622 | T | C |
|  | Chr5_28738632 | C- | TT |
|  | Chr5_28738638 | A | G |
|  | Chr5_28738653 | T | C |
|  | Chr5_28738686 | T | C |

**Table S4. (Continued)**

| **Gene Name** | **Position** | **93-11** | **Wild rice** |
| --- | --- | --- | --- |
| *LOC_Os05g48810* | Chr5_28738702 | T | C |
|  | Chr5_28738719 | CG | TA |
|  | Chr5_28738848 | C | T |
|  | Chr5_28739036 | G | A |
|  | Chr5_28739190 | C | T |
|  | Chr5_28739284 | T | G |
|  | Chr5_28739316 | G | A |
| *LOC_Os05g48820* | Chr5_28740184 | G | A |
|  | Chr5_28740224 | T | C |
|  | Chr5_28741768 | A | T |
|  | Chr5_28743265 | A | G |
|  | Chr5_28743295 | G | A |
|  | Chr5_28747963 | A | G |
|  | Chr5_28749159 | A | G |
|  | Chr5_28749459 | T | C |

**Table S4. (Continued)**

| **Gene Name** | **Position** | **93-11** | **Wild rice** |
| --- | --- | --- | --- |
| *LOC_Os05g48820* | Chr5_28750070 | A | C |
|  | Chr5_28750164 | G | A |
|  | Chr5_28750492 | - | C |
|  | Chr5_28750530 | G | A |
|  | Chr5_28750673 | G | C |
|  | Chr5_28751073 | A | G |
|  | Chr5_28751083 | C | A |
|  | Chr5_28751183 | T | C |
|  | Chr5_28751217 | T | G |
|  | Chr5_28751240 | C | T |
|  | Chr5_28751288 | CC | TT |
|  | Chr5_28751309 | T | C |
|  | Chr5_28751334 | CC | TT |
|  | Chr5_28751433 | A | T |
|  | Chr5_28751435 | C | G |

**Table S4. (Continued)**

| **Gene Name** | **Position** | **93-11** | **Wild rice** |
| --- | --- | --- | --- |
| *LOC_Os05g48820* | Chr5_28751453 | - | T |
|  | Chr5_28751497 | T | A |
|  | Chr5_28751546 | C | T |
|  | Chr5_28751550 | G | A |
|  | Chr5_28751553 | A | G |
|  | Chr5_28751616 | --- | CAA |
|  | Chr5_28751693 | C | G |
|  | Chr5_28751707 | G | A |
|  | Chr5_28751819 | A | G |
|  | Chr5_28751834 | A | G |
|  | Chr5_28751889 | A | G |
|  | Chr5_28751921 | - | T |
|  | Chr5_28751923 | C | T |
|  | Chr5_28751929 | G | A |
|  | Chr5_28751958 | A | G |

**Table S4. (Continued)**

| **Gene Name** | **Position** | **93-11** | **Wild rice** |
| --- | --- | --- | --- |
| *LOC_Os05g48830* | Chr5_28751969 | C | T |
|  | Chr5_28751978 | T | C |
|  | Chr5_28751995 | T | C |
|  | Chr5_28752016 | T | C |
|  | Chr5_28752103 | T | C |
|  | Chr5_28752175 | T | C |
| *LOC_Os05g48840* | Chr5_28754856 | T | C |
|  | Chr5_28754905 | A | C |
|  | Chr5_28754920 | - | G |
|  | Chr5_28757195 | G | A |
|  | Chr5_28757347 | A | T |
|  | Chr5_28757393 | AAT | --- |
|  | Chr5_28757531 | C | T |
|  | Chr5_28757770 | A | G |
|  | Chr5_28757797 | G | C |

**Table S4. (Continued)**

| **Gene Name** | **Position** | **93-11** | **Wild rice** |
| --- | --- | --- | --- |
| *LOC_Os05g48840* | Chr5_28757846 | C | T |
|  | Chr5_28757921 | C | T |
|  | Chr5_28757984 | C | G |
|  | Chr5_28758034 | A | G |
| *LOC_Os05g48850* | Chr5_28780381 | G | A |
|  | Chr5_28780400 | ---- | AGGG |
|  | Chr5_28780435 | G | A |
|  | Chr5_28780464 | C | T |
|  | Chr5_28780469 | A | G |
|  | Chr5_28780561 | CCGCCA | -----G |
|  | Chr5_28780598 | C | T |
|  | Chr5_28780603 | T | C |
|  | Chr5_28780632 | A | G |
|  | Chr5_28780636 | TA | CT |
|  | Chr5_28780677 | T | C |

**Table S4. (Continued)**

| **Gene Name** | **Position** | **93-11** | **Wild rice** |
| --- | --- | --- | --- |
| *LOC_Os05g48850* | Chr5_28780741 | A | G |
|  | Chr5_28780749 | C | A |
|  | Chr5_28780779 | G | A |
|  | Chr5_28780825 | C | T |
|  | Chr5_28780880 | C | T |
|  | Chr5_28780890 | G | C |
|  | Chr5_28780975 | - | A |
|  | Chr5_28781709 | T | C |
|  | Chr5_28781758 | G | A |
|  | Chr5_28781769 | G | A |
|  | Chr5_28781785 | C | T |
|  | Chr5_28781818 | C | A |
|  | Chr5_28781837 | C | T |
|  | Chr5_28781903 | G | T |
|  | Chr5_28782005 | A | G |

**Table S4. (Continued)**

| **Gene Name** | **Position** | **93-11** | **Wild rice** |
| --- | --- | --- | --- |
| *LOC_Os05g48850* | Chr5_28782340 | - | T |
|  | Chr5_28782723 | T | C |
|  | Chr5_28783664 | ------ | GCCGCC |
| *LOC_Os05g48855* | Chr5_28784688 | A | G |
|  | Chr5_28784950 | A | G |
|  | Chr5_28785410 | A | G |
|  | Chr5_28785559 | AG | GC |
|  | Chr5_28785637 | C | T |
|  | Chr5_28785699 | T | C |
|  | Chr5_28786129 | G | A |
|  | Chr5_28786250 | C | T |
|  | Chr5_28786439 | A | G |
|  | Chr5_28786444 | C | A |
|  | Chr5_28786531 | G | T |
|  | Chr5_28786923 | T | C |

**Table S4. (Continued)**

| **Gene Name** | **Position** | **93-11** | **Wild rice** |
| --- | --- | --- | --- |
| *LOC_Os05g48855* | Chr5_28786439 | G | A |
|  | Chr5_28787135 | A | G |
|  | Chr5_28787153 | C | T |

Note：Sequence variations in the promoter and coding regions of the 12 genes are marked in red and black, respectively.

**Table S5. Information of 18 genes identified by analysis of RNA-seq and ChIP-seq data**

| **Gene Name** | **Description** |
| --- | --- |
| *LOC_Os11g05650* | mRNA-decapping enzyme, putative, expressed |
| *LOC_Os02g03410* | CAMK_CAMK_like.12 - CAMK includes calcium/calmodulin depedent protein kinases, expressed |
| *LOC_Os02g18080* | NB-ARC domain containing protein, expressed |
| *LOC_Os02g48980* | GPI-anchored protein, putative, expressed |
| *LOC_Os03g25490* | cytochrome P450 72A1, putative, expressed |
| *LOC_Os03g57310* | syntaxin, putative, expressed |
| *LOC_Os03g61120* | anthranilate synthase component I-1, chloroplast precursor, putative, expressed |
| *LOC_Os04g44750* | transporter family protein, putative, expressed |
| *LOC_Os04g52640* | SHR5-receptor-like kinase, putative, expressed |
| *LOC_Os05g30790* | CW-type Zinc Finger, putative, expressed |
| *LOC_Os06g15910* | potassium transporter, putative, expressed |
| *LOC_Os06g48950* | auxin response factor 19, putative, expressed |
| *LOC_Os07g29330* | serine/threonine-protein kinase CTR1, putative, expressed |
| *LOC_Os08g09060* | Cupin domain containing protein, expressed |
| *LOC_Os08g39560* | OTU-like cysteine protease family protein, putative, expressed |

**Table S5. (Continued)**

| **Gene Name** | **Description** |
| --- | --- |
| *LOC_Os09g09000* | transposon protein, putative, CACTA, En/Spm sub-class, expressed |
| *LOC_Os09g09930* | heavy metal transport/detoxification protein, putative, expressed |
| *LOC_Os09g27080* | growth regulator related protein, putative, expressed |

Note：Seven of the 18 genes that have been functionally characterized are indicated in red font.

**Table S6. Primers information used in this study**

| **Primer** | **Sequence (5'-3')** |
| --- | --- |
| ST5-CDS-F | ATGGACTCGGAGCACTG |
| ST5-CDS-R | TTAATCTCCAAATAAAGTAGTGAGCAG |
| ST5-Pro-F | GGAGATCCTGACCATCGATGTGAAG |
| ST5-Pro-R | AGGCTTCGGCGGAGCGGGTAAG |
| ST5-KO-F | GGGGAGCGAGCCTTATCT |
| ST5-KO-R | TTGCCAACATTTCCACTTCT |
| ST5-OE-F | TTTCATTTGGAGAGAACACGGGGGACTTT |
| ST5-OE-R | TTATCATCATCATCTTTATAATCAATGTCGTGGTCTTTGTAG |
| OsWRKY80-KO-F | ATGGCCAGCAGTAGCG |
| OsWRKY80-KO-R | CGTGCTTGCGCACCGAACACC |
| ACTIN-F | TCAGCAACTGGGATGATATGGAG |
| ACTIN-R | GCCGTTGTGGTGAATGAGTAAC |
| QST5-F | GTTATTCCAGGCAGTC |
| QST5-R | ACAGAAGAGGGTCAGC |
| QOsCPK4-F | GCCAGGCATCAGTTCAGGT |

**Table S6. (Continued)**

| **Primer** | **Sequence (5'-3')** |
| --- | --- |
| QOsCPK4-R | GACGCATGTTGGACAATACAG |
| GFP-ST5-F | TACCCGGGCTCTAGAATGGACTCGGAGCACTG |
| GFP-ST5-R | CAAATCGACTCTAGACTCCAAATAAAGTAGTGAGCAGC |
| 62SK-OsWRKY80-F | CGCTCTAGAACTAGTGGATCCATGGCCAGCAGTAGCG |
| 62SK-OsWRKY80-R | GTCGACGGTATCGATAAGCTTTTACATCTGAGGTCCAAATGATGGAGGCCTG |
| 62SK-ST5-F | CGCTCTAGAACTAGTGGATCCATGGACTCGGAGCACTG |
| 62SK-ST5-R | GTCGACGGTATCGATAAGCTTTTAATCTCCAAATAAAGTAGTGAGCAG |
| 0800-ST5-F | GTCGACGGTATCGATAAGCTTGGAGATCCTGACCATCGATGTGAAG |
| 0800-ST5-R | CGCTCTAGAACTAGTGGATCCAGGCTTCGGCGGAGCGGGTAAG |
| 0800-OsCPK4-F | GTCGACGGTATCGATAAGCTTACACATTTTATACTAGTGATTGTAGTG |
| 0800-OsCPK4-R | CGCTCTAGAACTAGTGGATCCGGGGGAGGAGGAGGGTTTG |
| B42AD-OsWRKY80-F | TGCCTCTCCCGAATTCATGGCCAGCAGTAGCG |
| B42AD-OsWRKY80-R | CGAGTCGGCCGAATTCCATCTGAGGTCCAAATGATGGAGGCCTG |
| Lacz-ST5-F | AAATGATGAATTGAAAAGCTTTTGGGTTAGAGGTAGGGTTGCA |
| Lacz-ST5-R | GTCGACAGATCCCCGGGTACCCATTCTTTAACCCACTCCTAAATTAAACCCTC |

**Table S6. (Continued)**

| **Primer** | **Sequence (5'-3')** |
| --- | --- |
| MBP-OsWRKY80-F | GAGGGAAGGATTTCAGAATTCATGGCCAGCAGTAGCG |
| MBP-OsWRKY80-R | CAAGCTTGCCTGCAGGTCGACCATCTGAGGTCCAAATGATGGAGGCCTG |
| Biotin-9311-3W-box-F | TAATTTGACCTAAAAATAGTATATATTGACCCACTTGTAAAAAAATAAAAATGAGTATATAAAAAAACACTTGCATATTTAGATACGGTTATCATCTAGTATTTGACCTATA |
| Biotin-9311-3W-box-R | TATAGGTCAAATACTAGATGATAACCGTATCTAAATATGCAAGTGTTTTTTTATATACTCATTTTTATTTTTTTACAAGTGGGTCAATATATACTATTTTTAGGTCAAATTA |
| Biotin-NIL-5W-box-F | TAATTTGACCTAAAAATGAGTATATATTGACCCACTTGCATATTGACCTAAAAATGAGTATATATTGACCCACTTGCATATTTAGATACGGTTATCATCTAGTATTTGACCTATA |
| Biotin-NIL-5W-box-R | TATAGGTCAAATACTAGATGATAACCGTATCTAAATATGCAAGTGGGTCAATATATACTCATTTTTAGGTCAATATGCAAGTGGGTCAATATATACTCATTTTTAGGTCAAATTA |
| MBP-ST5-F | GAGGGAAGGATTTCAGAATTCATGGACTCGGAGCACTG |
| MBP-ST5-R | CAAGCTTGCCTGCAGGTCGACATCTCCAAATAAAGTAGTGAGCAG |
| Biotin-OsCPK4-probe-F | ATCTAAATTGTAGTAGCGTG |
| Biotin-OsCPK4-probe-R | CACGCTACTACAATTTAGAT |
| Biotin-OsCPK4-mutant-probe-F | ATCTAACCCCCCGTAGCGTG |
| Biotin-OsCPK4-mutant-probe-R | CACGCTACGGGGGGTTAGAT |

**Table S6. (Continued)**

| **Primer** | **Sequence (5'-3')** |
| --- | --- |
| QOsCPK4-F1 | ACATTTTATACTAGTGATTGT |
| QOsCPK4-R1 | GATTTCTTCCAACTCACTATC |
| QOsCPK4-F2 | ACTCATTGCTACGAAACTGAAT |
| QOsCPK4-R2 | TACTCCGTTCAATAATCTATG |
| QOsCPK4-F3 | GCTACACGTACTAATTTTTTCTTAC |
| QOsCPK4-R3 | GCTACCCATGCTAAATTTTTC |

Note：Highlighted areas represent W-box.
